# Abscisic Acid and Calcium Signals Convergently Regulate Sugar Accumulation by Orchestrating the SRK2A/CIPK6‐ABI5‐TST2 Module in Citrus

**DOI:** 10.1111/pbi.70341

**Published:** 2025-09-09

**Authors:** Zuolin Mao, Mengdi Li, Siyang Gao, Zeqi Zhao, Xiawan Zhai, Ji‐Hong Liu, Lailiang Cheng, Chunlong Li

**Affiliations:** ^1^ National Key Laboratory for Germplasm Innovation & Utilization of Horticultural Crops, College of Horticulture and Forestry Sciences Huazhong Agricultural University Wuhan China; ^2^ Hubei Hongshan Laboratory Wuhan China; ^3^ Horticulture Section, School of Integrative Plant Science Cornell University Ithaca New York USA

**Keywords:** abscisic acid, calcium, *citrus*, sugar transporter, transcriptional regulation

## Abstract

Abscisic acid (ABA) and calcium respectively work as crucial plant hormones and second signalling molecules in the regulation of fruit development and quality formation, including the sugar content and flavour quality. However, the regulatory mechanisms of fruit sugar accumulation arising from the interaction between ABA and calcium have not yet been fully elucidated. Here, we show that the application of ABA or calcium enhances sugar accumulation in sweet orange ( *Citrus sinensis* ) fruit, accompanied by upregulation of the expression level of *tonoplast sugar transporter 2* (*CsTST2*), which mediates the transport of sugars into the vacuole. Based on molecular and transgenic analyses, we identified CsABI5 as the core transcription factor that positively regulates *CsTST2* expression for sugar accumulation. Both CsSRK2A, a key player in ABA signalling, and CsCIPK6, a kinase linked to calcium signalling, interact with and phosphorylate CsABI5 at distinct amino acid sites to enhance its transcriptional activity. Interestingly, CsABI5 also activates the expression of *CsCIPK6* by binding to its promoter, thereby establishing a positive feedback loop between ABA and calcium signalling that cascades into higher expression of *CsTST2* and sugar content. Collectively, these findings uncover a novel molecular network that underlies sugar accumulation in citrus, revealing the intricate coordination between ABA and calcium signalling in regulating tonoplast sugar transport in a non‐climacteric fruit.

## 1 Introduction

Sugars play a key role in determining the taste and flavour of fleshy fruits (Fang et al. 2023). The total sugar content in fruits is predominantly determined by the concentration of soluble sugars within vacuoles (Ren et al. 2023), with their transport from the cytosol into the vacuolar compartment mediated by tonoplast sugar transporters (Hedrich et al. 2015; Liu et al. 2023). For example, the apple tonoplast‐localised sugar transporters, MdERDL6 and MdTSTs, function synergistically to enhance fruit sugar accumulation (Zhu et al. 2021). In addition, in sugar beet (Jung et al. 2015; Okooboh et al. 2022) and watermelon (Ren et al. 2018, 2021), the high‐level expression of TSTs also exhibits a close correlation with the accumulation of soluble sugars. By conducting proteomic analysis of citrus vacuole, we have identified a close link between the protein and mRNA levels of *CsTST2* of sweet orange and the accumulation of soluble sugars during fruit development (Li et al. 2024; Mao et al. 2024). Although the role of TSTs in the accumulation of sugars is widely recognised, their transcriptional regulation during fruit development or in response to environmental factors remains elusive.

Abscisic acid (ABA) is an important phytohormone regulating plant growth and stress response. The ABA signal transduction pathway comprises the PYR/PYL/RCAR receptors, PP2C protein phosphatases, the SNF1‐related protein kinase 2 (SnRK2) and ABF/AREB transcription factors (TFs) (Chen et al. 2020; Soma et al. 2020; Lin et al. 2021). ABA has been recognised for its vital role in plant responses to environmental stresses (Li, Li, et al. 2020; Guo et al. 2021; Vonapartis et al. 2022). Moreover, recent research has increasingly focused on the role of ABA in the regulation of fruit quality. For instance, ABA promotes flesh softening during the ripening process of mango and kiwifruit (Huang et al. 2021; Wu et al. 2022). SlMYB99 acts as a downstream regulator of ABA signal that actively facilitates the synthesis and accumulation of ascorbic acid in tomato fruit (Xu, Huang, et al. 2023). In non‐climacteric fruits, such as strawberries, citrus and grapes, ABA serves as a primary hormone that regulates the processes of fruit ripening and the formation of quality attributes (Jia et al. 2013; Bai et al. 2021; Li et al. 2022). In citrus fruit, the ABA level increases during fruit development and plays a crucial role in regulating carotenoid metabolism within the pericarp, as well as determining fruit size (Feng et al. 2021; Sun, He, Feng, et al. 2024; Sun, He, Wei, et al. 2024; Zhang, Xu, et al. 2024). However, the role of ABA in the accumulation of soluble sugars, a critical aspect of citrus fruit ripening and quality formation, has not been thoroughly elucidated.

Calcium (Ca^2+^) is not only an important mineral nutrient in plants, but also acts as an intracellular secondary messenger for regulating plant growth, development and environmental responses (Tian et al. 2020; Wang et al. 2023). The regulatory function of calcium relies on the well‐documented calcium signalling decoders, including calmodulin (CaM), calmodulin‐like (CML) proteins, calcineurin B‐like (CBL) proteins coupled with their interacting kinases (CIPKs) and calcium‐dependent protein kinases (CDPKs) (Kudla et al. 2018; Tian et al. 2020). These components have long been acknowledged for their roles in mediating responses to both biotic and abiotic stresses and modulating mineral uptake and homeostasis (Luan and Wang 2021; Li et al. 2023). In addition, recent studies have revealed that calcium plays a pivotal role in the process of fruit ripening and the formation of various quality attributes. For example, the phosphorylation of MdACO1 by MdCDPK7 promotes its degradation and decreases ethylene synthesis, leading to delayed ripening of apple (Xu, Liu, et al. 2023). CaCIPK13 mediates the accumulation of anthocyanins in chilli peppers in response to low temperature (Ma et al. 2022). Very recently, CDPK26/27 act as sugar brakes by phosphorylating a sucrose synthase in tomato (Zhang, Lyu, et al. 2024). The level of calcium increases in citrus fruit at the late stages of development (Zhou et al. 2018). However, the role and regulation mechanisms of calcium signalling in the sugar accumulation of citrus fruit remain largely unclear.

Previous reports demonstrated that ABA and calcium signalling pathways interact in response to environmental stressors (Diaz et al. 2016; Edel and Kudla 2016; You et al. 2023). In rice (*Oryza sativa L*), Arabidopsis ( *Arabidopsis thaliana* ) and apple ( *Malus pumila* Mill.), the increase in plant drought tolerance through ABA is mediated by calcium signalling‐dependent mechanisms (Kim et al. 2010; Ma et al. 2017; Ni et al. 2019; Yang et al. 2024). In tomato, knocking out *CPK27* impairs ABA‐orchestrated plant responses to drought stress (Zhu, Jing, et al. 2024). Similarly, AtCPK4 and AtCPK11 augment ABA signalling through phosphorylation of AtABF1 and AtABF4, thereby contributing to salt tolerance in *Arabidopsis* (Zhu et al. 2007). Crop quality is often inextricably associated with environmental stress. Conditions such as drought and low temperatures frequently alter the processes of ripening and metabolite accumulation, which are dependent on ABA and calcium signalling pathways (Luang et al. 2018; Xiong et al. 2021). For instance, the application of exogenous ABA enhances calcium uptake into tomato fruit, thereby preventing blossom end rot (BER) (de Freitas et al. 2011). In papayas, the calcium sensor CML15 regulates fruit ripening through its interaction with PP2C46/65 (Zhu, Tan, et al. 2024). The accumulation of soluble sugars in citrus fruit is accompanied by an increase in ABA and calcium levels (Zhou et al. 2018; Feng et al. 2021). However, it is yet to be established whether and how these two signalling pathways interact to enhance the accumulation of soluble sugars.

In this study, we aimed to uncover the molecular network that governs how ABA and calcium regulate sugar accumulation during citrus fruit development, with a particular emphasis on the transcriptional regulation of *CsTST2*. We identified and characterised CsABI5, a TF crucial for sustaining the expression of *CsTST2* and two kinases that phosphorylate CsABI5: CsSRK2A, a key player in ABA signalling, and CsCIPK6, a kinase linked to calcium signalling. In addition, we found that CsABI5 regulates the expression of *CsCIPK6*, demonstrating an intricate relationship between ABA and calcium signalling pathways during sugar accumulation. These findings reveal a novel regulatory mechanism that underlies sugar accumulation in citrus, a non‐climacteric fruit.

## 2 Results

### 2.1 ABA and Calcium Promote Sugar Accumulation by Upregulating CsTST2 Expression in Citrus Fruit

**FIGURE 1:**
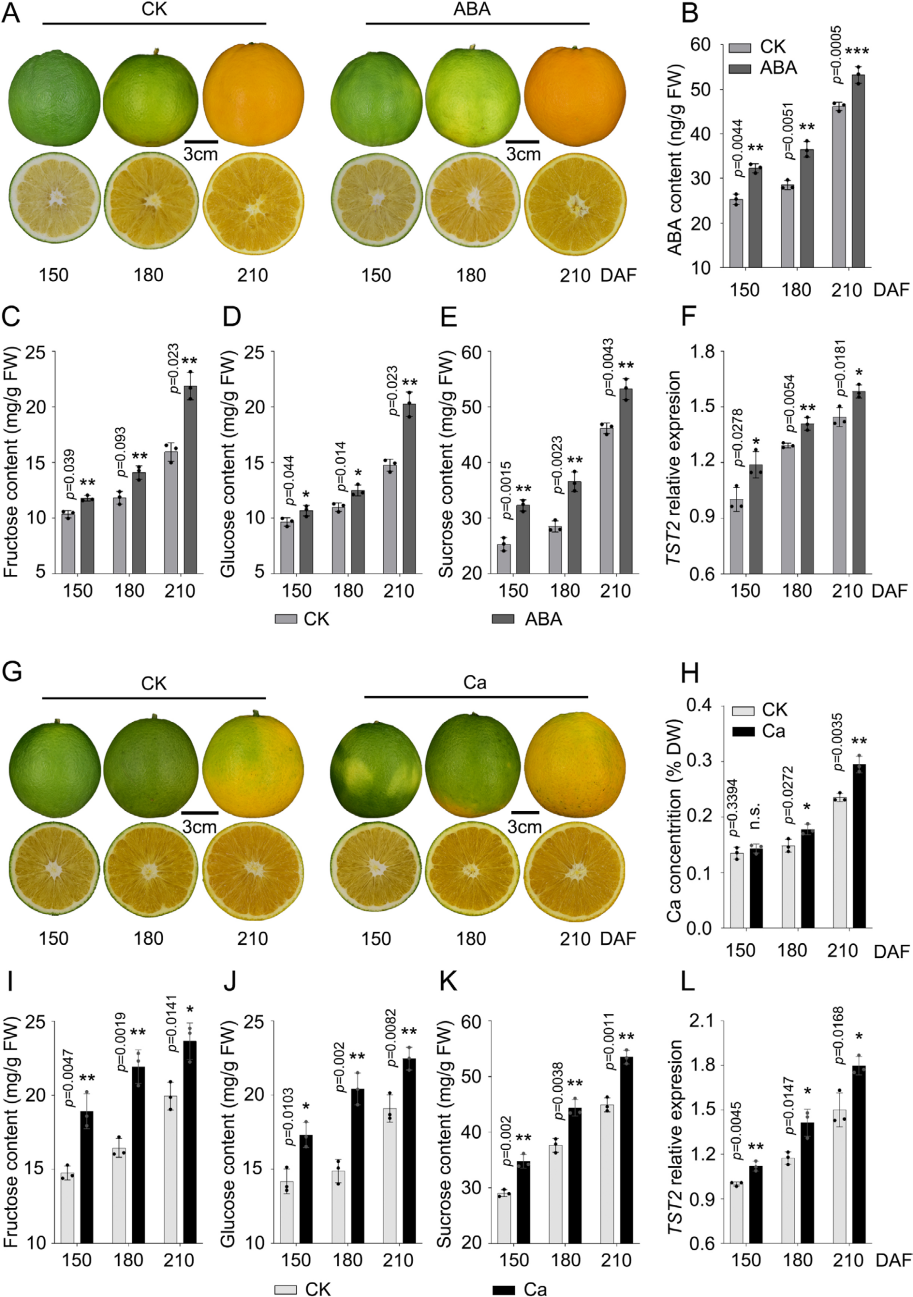
ABA and calcium increase sugar content and *CsTST2* expression in sweet orange fruit. (A–F) In both ABA‐treated and control pulp (A), ABA content was quantified using high‐performance liquid chromatography coupled with tandem mass spectrometry (HPLC–MS/MS) (B), and the fructose (C), glucose (D) and sucrose (E) levels and the expression level of *CsTST2* (F) were detected using gas chromatography (GC) and quantitative reverse transcription polymerase chain reaction (RT‐qPCR), respectively. (G–L) Ca‐treated and control pulp (G), Ca content was evaluated by using an inductively coupled plasma optical emission spectrometer (ICP‐OES) (H), and the fructose (I), glucose (J) and sucrose (K) levels and the expression level of *CsTST2* (L) were detected using GC and RT‐qPCR, respectively. The *CsTST2* transcript level was set to ‘1’ in the ABA‐ and Ca‐treated controls at 150 DAF. Nine fruits from a specific treatment in each period were used as an independent biological replicate. Three biological replicates were analysed. Values are means ± standard deviation (SD). Statistical significance was determined using two‐tailed Student's *t*‐test (*p < 0.05, **p < 0.01, ***p < 0.001). n.s., no significant difference. DAF, days after flowering.

To investigate the effects of ABA and calcium on sugar accumulation in citrus, we treated the *Citrus sinensis* fruits starting at 120 days after flowering (DAF) with 100 μM ABA or 100 mM calcium nitrate at weekly intervals and collected fruit samples at 150, 180 and 210 DAF, respectively (Figure 1A,G). These two treatments significantly increased fruit ABA or calcium concentrations (Figure 1B,H), leading to more accumulation of fructose, glucose and sucrose in the fruit (Figure 1C–E,I–K). In earlier work, we confirmed the crucial role of CsTST2 in sugar accumulation in citrus fruit (Li et al. 2024; Mao et al. 2024). The transcript level of *CsTST2* was significantly elevated by ABA or calcium treatment relative to the control (Figure 1F,H). These results indicate that ABA and calcium enhance sugar accumulation by upregulating *CsTST2* expression in citrus fruit, which prompted us to explore the transcriptional regulation of *CsTST2* underlying these responses.

### 2.2 CsABI5 Enhances the Expression of *CsTST2* by Directly Binding to Its Promoter

**FIGURE 2:**
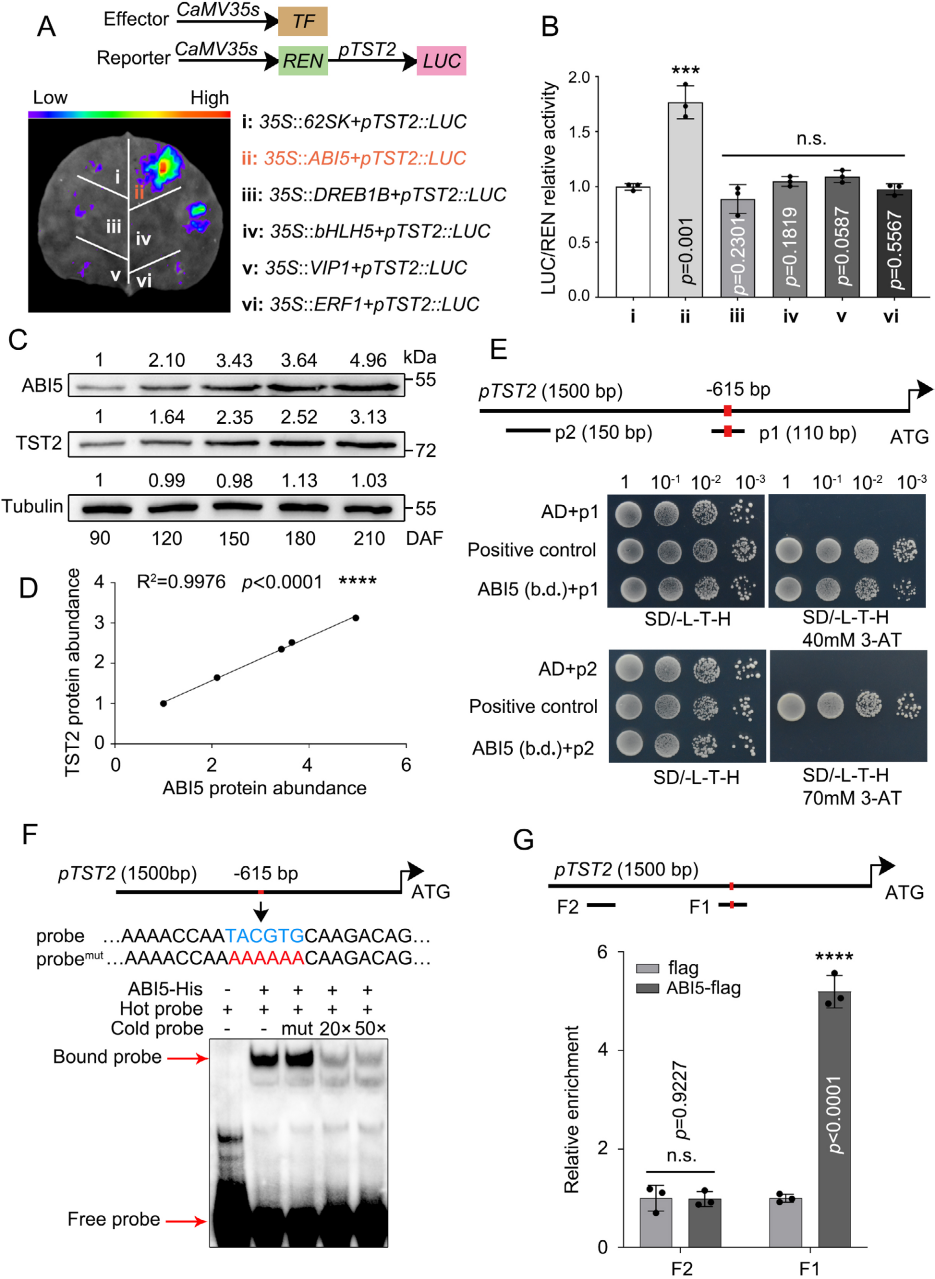
CsABI5 is a positive transcription factor regulating *CsTST2* expression. (A) The dual luciferase activation analysis was conducted in *N. benthamiana* leaves. Amongst the five transcription factors co‐expressed with *CsTST2*, *CsABI5*, *CsDREB1B*, *CsbHLH5*, *CsVIP1* and *CsERF1*, *CsABI5* emerged as the most promising candidate for activating *CsTST2* expression. (B) The LUC/REN relative enzyme activity analysis demonstrated that *CsABI5* exhibited a greater transcriptional activation capacity for the promoter of *CsTST2* (*pTST2*). (C) The protein levels of CsABI5 and CsTST2 were quantified in the pulp of sweet oranges by using specific antibodies anti‐CsABI5 and ‐CsTST2. The tubulin protein works as an internal standard to ensure consistent protein loading. Samples were collected at 30‐day intervals from 90 to 210 DAF for five periods. (D) Quantitative analysis of protein expression, conducted using Image J (version 1.4.3.67), demonstrated that CsABI5 and CsTST2 were well correlated at the protein level (*r* ^2^ = 0.9976, ****p < 0.0001). (E) Y1H analysis showed that the DNA‐binding domain of CsABI5 (b.d.) could bind to the *pCsTST2* fragment p1 with the ABRE element, whilst it does not bind to the *pCsTST2* fragment p2 without the ABRE element. The red rectangle represents the ABRE element. Each colony was diluted in 50 μL of sterile water, subjected to a gradient dilution and incubated in a specific medium for a duration of 3–4 days. (F) EMSA analysis demonstrated that CsABI5 binds to the ABRE element within the *CsTST2* promoter in vitro. The biotin‐free labelled probe was used as a competitor, and mutation of the ABRE element (probe^mut^) resulted in the loss of competitive effect of the cold probe. The red rectangles illustrate DNA probes that contain the ABRE element. (G) ChIP‐qPCR assay demonstrates that CsABI5 binds to the promoter of *CsTST2* in vivo. ChIP‐qPCR was employed to assess the degree of enrichment of F1 containing the ABRE element and F2 lacking the ABRE element. The enrichment degree of fragment in Flag control calli was set to ‘1’, and red rectangles represent the ABRE element. Bars represent the mean ± SD (*n* = 3 independent biological replicates). The asterisks indicate significant differences as assessed by two‐tailed Student's *t*‐test (***p < 0.001, ****p < 0.0001). n.s., not significant.

To identify the TF for regulating *CsTST2* expression during citrus fruit development and sugar accumulation, we conducted a Short Time‐series Expression Miner (STEM) cluster analysis (Ernst and Bar‐Joseph 2006) based on Fragment Per Kilobase of exon per Million mapped reads (FPKM) values derived from previously reported transcriptome data (Feng et al. 2021). A group of 113 co‐expressed genes within the expression module were associated with *CsTST2* (Figure S1A and Table S1), amongst which, five genes, *CsABI5*, *CsDREB1B*, *CsbHLH5*, *CsVIP1* and *CsERF*,*1* were identified as TFs according to Gene Ontology (GO) annotation (Table S1). To evaluate their relationship with *CsTST2* expression, we performed a dual luciferase (Luc) assay in *Nicotiana benthamiana* leaves using the 35S promoter to drive their CDSs as an effector alongside p*CsTST2*::LUC as a reporter. Co‐expressing 35S::*CsABI5* with p*CsTST2*::LUC resulted in dramatically stronger LUC fluorescence and higher relative LUC activity compared with the control and other combinations (Figure 2A,B). CsABI5 was confirmed to localise to the nucleus, suggesting that CsABI5 may function as a TF regulating the expression of *CsTST2* by binding to the putative ABA‐responsive element (ABRE) in the *CsTST2* promoter (p*CsTST2*) sequence (Figures S1B and S2A). A significant correlation at both the transcriptional and protein (using each specific antibodies) levels between CsABI5 and CsTST2 to further enhance the reliability of this hypothesis (Figure 2C,D and Figure S3).

We subsequently conducted yeast one‐hybrid (Y1H), electrophoretic mobility shift assay (EMSA) and chromatin immunoprecipitation (ChIP) experiments to verify the binding of CsABI5 to the promoter of *CsTST2*. For the Y1H experiment, the DNA‐binding domain (b.d.) of *CsABI5* was cloned into the pGADT7 vector (Figure S2B), and the p*CsTST2* fragment with (p1) or without (p2) the ABRE element was inserted into the pHIS2. The yeast growth results showed that the transformant with the combination of the fragment containing the ABRE element grew normally on SD/−L/−T/−H medium containing 40 or 70 mM 3‐amino‐1,2,4‐triazole (3‐AT), whilst the fragment without the ABRE element or the pGADT7 empty vector combination failed to grow (Figure 2E), indicating that CsABI5 binds to the ABRE element of p*CsTST2*. Further, the EMSA experiment involving the CsABI5‐His fusion protein and a biotin‐labelled p*CsTST2* DNA probe containing the ABRE element demonstrated that the CsABI5 protein specifically binds to the ABRE element in the *CsTST2* promoter in vitro, whereas a cold probe with the ABRE element mutated lost its ability to compete with the biotin‐labelled probe for binding as the regular cold probe did (Figure 2F). Finally, we extracted cross‐linked DNA‐chromatin complexes from transgenic citrus calli expressing 35S::*CsABI5*‐3 × Flag for the ChIP‐PCR assay and found that the immunoprecipitated CsABI5 protein resulted in a higher enrichment of the p*CsTST2* fragment containing the ABRE element (F1), but not the fragment without the ABRE sequence (F2) (Figure 2G). Collectively, these findings indicate that CsABI5 specifically binds to the ABRE element in the *CsTST2* promoter, enhancing the transcriptional activity of *CsTST2* observed in the LUC assay.

### 2.3 CsABI5 Promotes Sugar Accumulation Through Upregulation of *CsTST2* Expression

**FIGURE 3:**
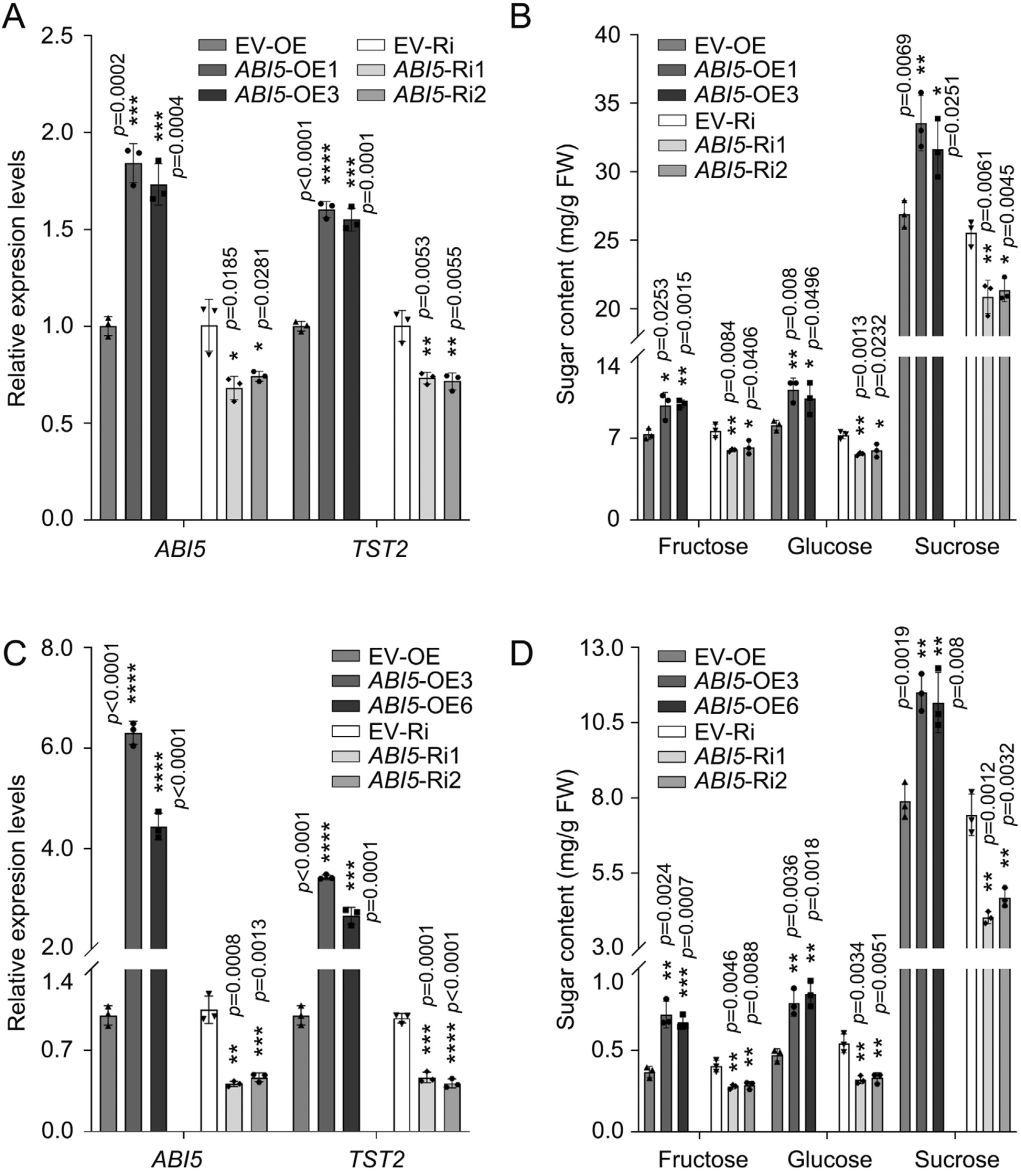
CsABI5 enhanced the sugar accumulation by upregulation of *CsTST2* expression. (A, B) The transient overexpression or silence of *CsABI5* in citrus juice sacs resulted in elevated or decreased transcript levels of *CgTST2* (A), as well as increased or reduced soluble sugar concentrations (B). The imbibed juice sacs were incubated on MS medium for a duration of 7 days, shielded from light, prior to analysis. All healthy sap sacs present on one dish were utilized as an independent biological replicate. (C, D) The alterations in the transcript level of *CsABI5* corresponded with simultaneous increases or decreases in the expression level of *CsTST2* (C), as well as variations in sugar content in the calli (D). Transgenic calli samples were collected at 21 days intervals, serving as independent biological replicates. Bars represent mean ± SD (*n* = 3 independent biological replicates). Asterisks indicate significant differences by two‐tailed Student's *t*‐test (*p < 0.05, **p < 0.01, ***p < 0.001, ****p < 0.0001).

To determine the physiological function of CsABI5, we overexpressed or silenced *CsABI5* in the juice sacs of *Citrus grandis* fruits utilising an *Agrobacterium*‐mediated method. This resulted in elevated or decreased expression of *CgTST2* and levels of sugar accumulation, respectively (Figure 3A,B). To validate the function of CsABI5, we stably transformed the *CsABI5*‐pH7WGD recombinant vector (for overexpression) into citrus calli. The overexpression of *CsABI5* resulted in higher expression levels of *CsTST2* and more accumulation of fructose, glucose and sucrose (Figure 3C,D). In contrast, the expression of *CsABI5* and *CsTST2* in *CsABI5* RNA interference (*ABI5*‐Ri) transgenic calli was decreased to approximately 50% of their initial levels, leading to a reduction in fructose, glucose and sucrose levels (Figure 3C,D). Based on our previous report (Li et al. 2024), the overexpression of *CsABI5* on the basis of *CsTST2* silencing (*TST2*‐Ri) failed to induce effective elevation of *CsTST2* expression level and soluble sugar content (Figure S4A,B), which indicates that the promotive effect of *CsABI5* on soluble sugar accumulation is dependent on *CsTST2* expression. These results indicate that CsABI5 promotes sugar accumulation in citrus by enhancing the transcript level of *CsTST2*.

### 2.4 Both CsCIPK6 and CsSRK2A Interact With CsABI5

**FIGURE 4:**
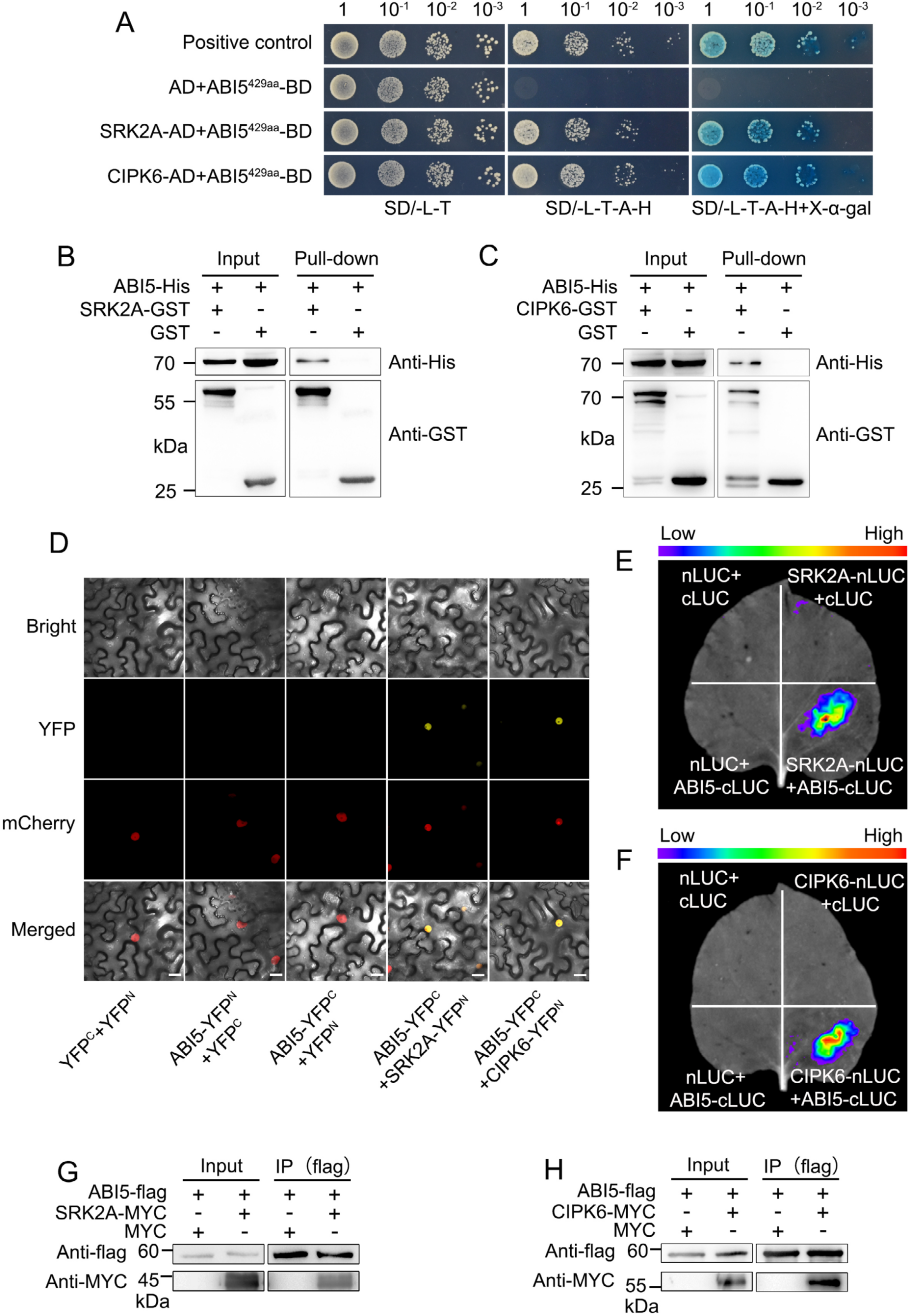
CsABI5 interacts with CsSRK2A and CsCIPK6. (A) In Y2H experiments, the *CsABI5* ^429aa^‐pGBKT7 recombinant vector was co‐transformed into Y2H yeast cells with *CsSRK2A*‐ and *CsCIPK6*‐pGADT7. The positive clones were able to grow and turn X‐α‐gal blue on SD/−A−H−L−T selection medium after dilution by gradient. In contrast, the transformant of the negative control (pGADT7) did not. (B, C) GST‐pull down experiments. CsABI5‐His protein was able to be co‐purified by CsSRK2A‐ and CsCIPK6‐GST, but not GST. (D) BiFC experiments. The YFP fluorescence signals were detected under a laser confocal microscope with the co‐expression of CsABI5‐YFPC and CsSRK2A‐ or CsCIPK6‐YFPN fusion proteins in *N. benthamiana* leaves, whereas YFP signals could not be detected for combinations containing YFPN or YFPC empty vector. Scale bars = 20 μm. (E, F) Luciferase complementation imaging assays. CDSs of *CsABI5*, *CsSRK2A* and *CsCIPK6* were inserted into vectors containing cLUC or nLUC. The fusion proteins were expressed in *N. benthamiana* leaves according to specific combinations mediated by *Agrobacterium* GV3101. Luciferase imaging showed that CsABI5 interacted with CsSRK2A and CsCIPK6, respectively. (G, H) Co‐IP analysis. CsABI5‐Flag was co‐expressed with CsSRK2A‐/CsCIPK6‐MYC fusion proteins in *N. benthamiana* leaves. Western blot analysis showed that CsSRK2A‐/CsCIPK6‐MYC could be co‐precipitated by CsABI5‐Flag. The above experiments were independently repeated three times with consistent results.

To explore how the CsABI5 transactivation activity is regulated for sugar accumulation, we carried out a yeast two‐hybrid (Y2H) screen using CsABI5‐BD bait protein within the self‐inactivation domain (*CsABI5* ^429aa^‐BD, Figure S5) on a cDNA library made from citrus fruit flesh. In total, 39 positive clones were detected, of which four were annotated as *CsSRK2A* and three were annotated as *CsCIPK6* (Table S2). The SnRK2 kinases are known for their involvement in ABA signalling, whereas CIPK kinases primarily perceive calcium signals transmitted by CBL proteins. Both SnRK2 and CIPK proteins have been shown to play crucial regulatory roles in various biological processes such as plant growth and development. We initially investigated the subcellular localization of the two candidates and found that both were localised to the cytoplasm and nucleus (Figure S6). The presence of the two proteins in the nucleus suggests a spatial possibility of interaction with CsABI5. Meanwhile, the reciprocal candidate CBL proteins of CsCIPK6 were screened through the Y2H analysis of the CsCBL family. Three strong interaction members, CsCBL1/4/5, were identified based on the yeast growth phenotype (Figure S7A). Considering that *CsCBL4* and *CsCBL5* exhibited negligible expression levels in citrus juice sacs based on previous data (Feng et al. 2021), we selected CsCBL1 for the next analysis. The CsCBL1‐CIPK6 interaction was further confirmed by Y2H point‐to‐point and LUC complementation imaging (LCI) assays (Figure S7B,C). Unlike previously reported membrane‐localisation of AtCBL1 (Batistic et al. 2008), CsCBL1 is localised to the cytoplasm and nucleus, even under the mutation of its N‐terminal (CsCBL1‐mut) into the conserved 12 amino acids of AtCBL1 (Figure S8). We concluded that a physical interaction exists between ABA or calcium signal transduction factors and CsABI5, prompting us to perform an in‐depth validation. The point‐to‐point Y2H assay demonstrated that yeast strains co‐transformed with *CsCIPK6*‐AD or *CsSRK2A*‐AD in conjunction with *CsABI5* ^429aa^‐BD exhibited normal growth on SD/−L/−T/−A/−H medium and turned blue in the medium containing X‐α‐gal compared to the control, indicating an interaction between them (Figure 4A). In vitro GST‐pull down demonstrated that the CsABI5‐His fusion protein was co‐purified with CsCIPK6‐ or CsSRK2A‐GST, but not with GST alone (Figure 4B,C). Subsequently, the strong yellow fluorescent protein (YFP) and firefly fluorescence signals further confirmed the interaction between CsABI5 and CsCIPK6 or CsSRK2A compared to their corresponding controls based on the bimolecular fluorescence complementation (BiFC) and LCI experiments (Figure 4D–F). Finally Co‐IP experiments using CsABI5‐Flag, CsSRK2A‐MYC or CsCIPK6‐MYC fusion protein expressed in *N. benthamiana* leaves show that both CsSRK2A and CsCIPK6 interact with CsABI5 in vivo (Figure 4G,H). Taken together, these findings show that CsSRK2A and CsCIPK6 interact with CsABI5, in response to ABA or calcium signal, respectively.

### 2.5 CsSRK2A and CsCIPK6 Phosphorylate CsABI5, Enhancing Its Transactivation Activity

**FIGURE 5:**
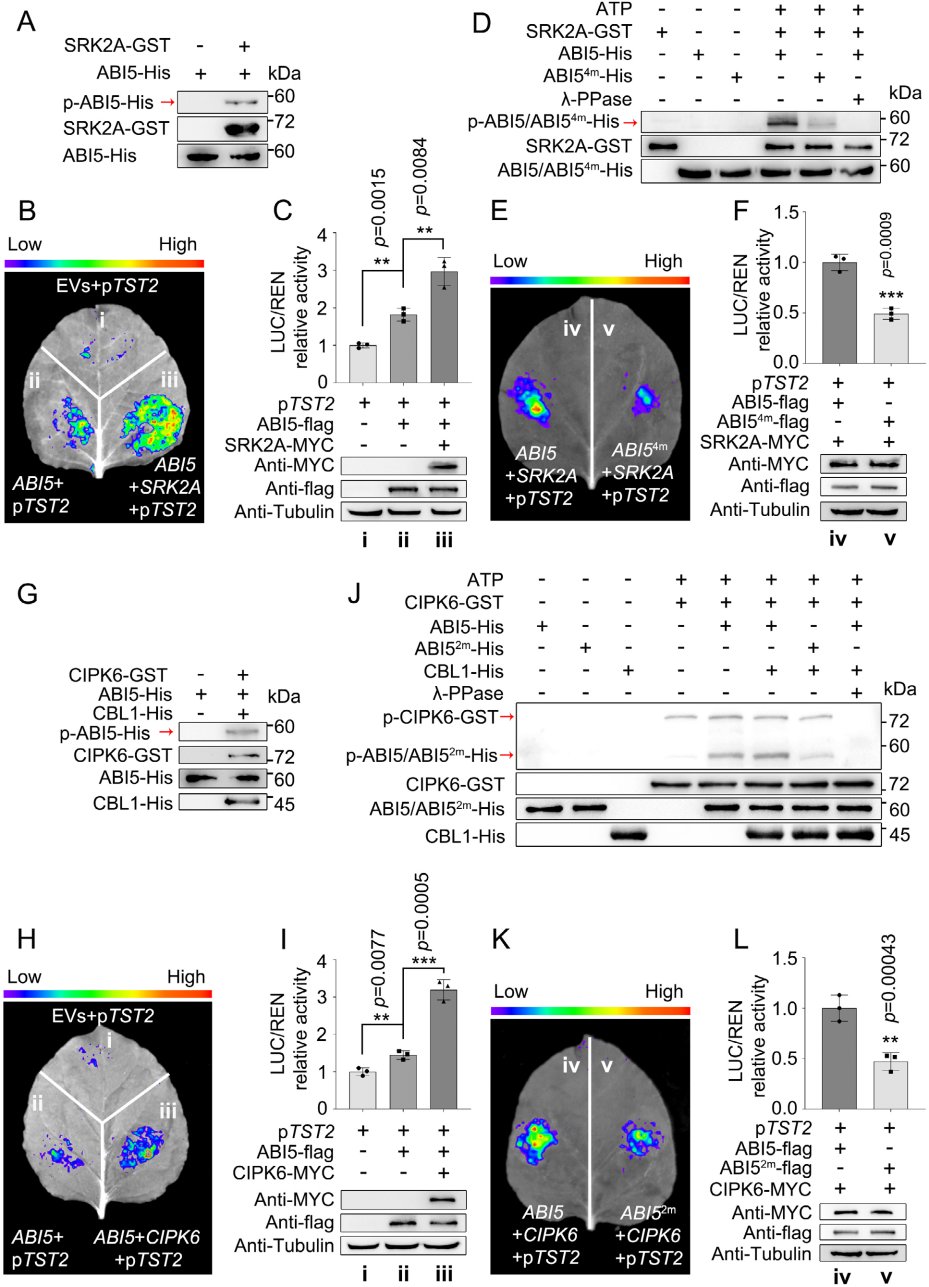
CsSRK2A and CsCIPK6 phosphorylate CsABI5 and enhance its transcriptional activation. (A) Western blot (WB) analysis involving anti‐pSer/pThr specific antibodies revealed that CsABI5 can be phosphorylated by CsSRK2A in vitro. (B, C) 35S::*CsABI5*, 35S::*CsSRK2A* were co‐expressed in *N. benthamiana* leaves according to a specific combination using LUC as a reporter gene. In vivo imaging (B) and relative enzyme activity assay (C) of firefly luciferase showed that, at a consistent concentration of the CsABI5 protein, CsSRK2A increased the transcriptional activation of CsABI5 to *pTST2*. WB involving anti‐Flag, ‐MYC and ‐tubulin antibodies were used for protein abundance analysis. Tubulin was used as an internal standard. (D) In vitro phosphorylation analysis revealed that Ala substitutions at positions 40, 109, 112 and 134 of CsABI5 (CsABI5^4m^) caused a substantial attenuation of the CsABI5^4m^ phosphorylation level by CsSRK2A, and this phosphorylation was subjected to a near‐complete elimination by λ‐PPase. (E, F) In vivo imaging (E) and relative enzyme activity assay (F) of firefly luciferase showed that the substitution of four phosphorylation sites in CsABI5 (CsABI5^4m^) impairs the enhancing effect of CsSRK2A on the transcriptional activation capacity of CsABI5. WB assays were performed to ensure that the protein concentrations were consistent on both sides, and tubulin was used as an internal control. (G) In vitro phosphorylation assays showed that CsABI5 could be phosphorylated by CsCIPK6 with the involvement of CsCBL1. (H, I) Dual luciferase in vivo imaging (H) and relative LUC activity analysis (I) showed that CsCIPK6 increased the transcriptional activation of CsABI5 to *pTST2*. WB assay ensured that the abundance of CsABI5 proteins on both sides was consistent. (J) In vitro phosphorylation analysis showed that CsCBL1 enhanced the phosphorylation effect of CsCIPK6 on CsABI5, but the substitution of Ala at positions 39 and 40 in CsABI5 (CsABI5^2m^) severely impaired the phosphorylation of CsABI5^2m^ by CsCIPK6. This phosphorylation was also subjected to an almost complete elimination by λ‐PPase. (K, L) In vivo imaging (K) and relative firefly luciferase activity assay (L) showed that the substitution of two phosphorylation sites in CsABI5 (CsABI5^2m^) reduced the potentiation of CsABI5 transcriptional activity by CsCIPK6. The WB assay was used to ensure consistency of protein concentrations on both sides of the leaf. Bars represent mean ± SD (*n* = 3 independent biological replicates). Asterisks indicate significant differences by two‐tailed Student's *t*‐test (**p < 0.01, ***p < 0.001).

Given CsSRK2A and CsCIPK6 are kinases, we performed phosphorylation assays on them. Both CsSRK2A and CsCIPK6 phosphorylated CsABI5 in vitro (Figure 5A,G), which suggests that it may exert more efficient regulatory functions in a phosphorylated form. Subsequent detection revealed that the phosphorylation level of CsABI5 was increased gradually with fruit development (Figure S9). To investigate the effects of CsSRK2A and CsCIPK6 on the transactivation activity of CsABI5, we co‐expressed 35S::*CsABI5*, 35S::*CsSRK2A*, 35S::*CsCIPK6*, 35S::Empty vector and *pCsTST2*::LUC in various combinations in *N. benthamiana* leaves and found that both CsSRK2A and CsCIPK6 effectively increased the transcriptional activation of *pCsTST2* by CsABI5 (Figure 5B,C,H,I). We then performed LC–MS/MS analysis to identify the phosphorylation sites of CsABI5 by CsSRK2A and CsCIPK6. CsSRK2A phosphorylated Ser40/Ser109/Thr112/Ser134 (Figure S10), while CsCIPK6 modified Ser39/Ser40 sites in the CsABI5 protein (Figure S11). The subsequent in vitro phosphorylation experiment revealed that, for CsCIPK6, the phosphorylation of CsABI5 was enhanced by the presence of CsCBL1, but the identified Ser/Thr to alanine (Ala) transition resulted in a substantially weakened phosphorylation level of CsABI5 by either CsCIPK6 or CsSRK2A. In addition, complete removal of λ‐PPase impeded the phosphorylation of CsABI5 by either CsCIPK6 or CsSRK2A (Figure 5D,J). To assess the importance of phosphorylation in the transactivation activity of CsABI5, we introduced mutations at the phosphorylation sites within CsABI5 that are targeted by CsSRK2A or CsCIPK6, substituting these sites with Ala rendered them non‐phosphorylatable. Following by co‐expression 35S::*CsABI5*, 35S::*CsABI5* with two mutated sites (35S::*CsABI5* ^*2m*^), 35S::*CsABI5* with four mutated sites (35S::*CsABI5* ^*4m*^), 35S::*CsSRK2A*, 35S::*CsCIPK6* and p*CsTST2*::LUC in *N. benthamiana* leaves according to specific combinations, LUC fluorescence and relative enzyme activity assays revealed that mutations in the phosphorylation sites substantially diminished the enhanced transcriptional activation of CsABI5 by CsSRK2A or CsCIPK6 (Figure 5E,F,K,L). These results indicate that CsSRK2A and CsCIPK6 facilitate the transcriptional activation of *CsTST2* through the phosphorylation of specific amino acid sites of CsABI5.

### 2.6 The Enhancement of Sugar Accumulation by CsCIPK6 and CsSRK2A is Dependent on the Phosphorylation of CsABI5

**FIGURE 6:**
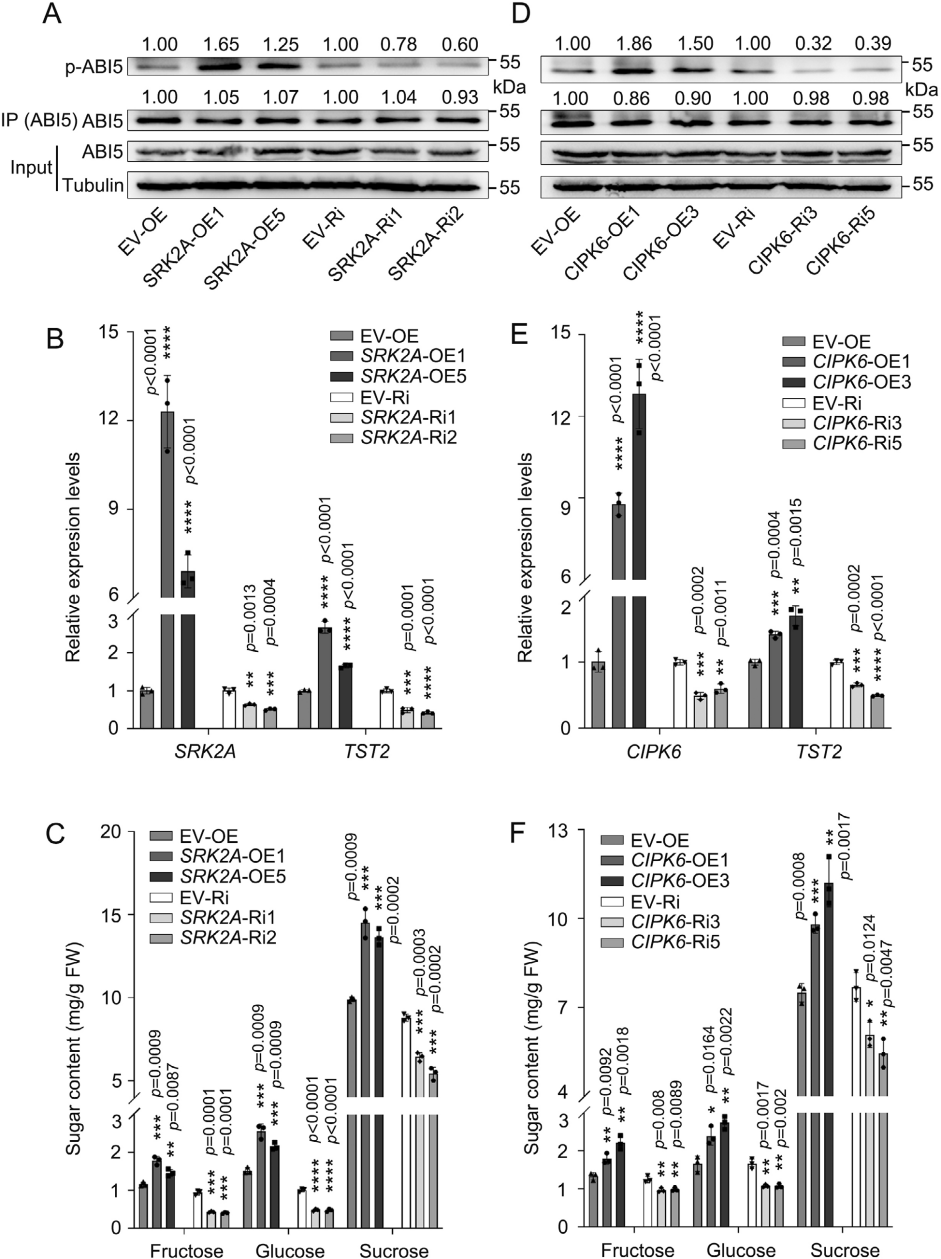
Effects of altered *CsSRK2A* or *CsCIPK6* transcript levels on CsABI5 phosphorylation, *CsTST2* expression and sugar content. (A) Detection of CsABI5 phosphorylation levels in *CsSRK2A* transgenic citrus calli. Empty vector‐transformed citrus calli were used as a control. CsABI5 protein was immunoprecipitated using an Anti‐CsABI5‐specific antibody. Immunoblot quantification was performed by Image J software (version 1.4.3.67). (B) *CsTST2* expression levels were assayed in *CsSRK2A* transgenic calli. The expression levels of the corresponding genes in empty vector‐transformed calli were set to ‘1’, and *CsActin* was used as an internal standard. (C) Fructose, glucose and sucrose contents were assayed in *CsSRK2A* transgenic citrus calli. (D) Detection of CsABI5 phosphorylation levels in *CsCIPK6* transgenic citrus calli. Empty vector‐transformed citrus calli were used as a control. CsABI5 protein was immunoprecipitated using an Anti‐CsABI5‐specific antibody. Immunoblot quantification was performed by Image J software (version 1.4.3.67). (E) *CsTST2* expression levels were assayed in *CsCIPK6* transgenic calli. The expression levels of the corresponding genes in empty vector‐transformed calli were set to ‘1’. *CsActin* was used as an internal standard. (F) Fructose, glucose and sucrose contents were assayed in *CsCIPK6* transgenic citrus calli. Transgenic calli samples were collected at 21‐day intervals, serving as independent biological replicates. Bars represent mean ± SD (*n* = 3 independent biological replicates). Asterisks indicate significant differences by two‐tailed Student's *t*‐test (*p < 0.05, **p < 0.01, ***p < 0.001, ****p < 0.0001).

To further confirm the roles of CsCIPK6 and CsSRK2A in the accumulation of soluble sugars, we overexpressed or silenced *SRK2A* or *CIPK6* in the juice sacs of citrus fruit and found a higher or lower accumulation of soluble sugars, respectively (Figure S12). We then generated overexpression and RNAi transgenic citrus calli to validate the physiological functions of these two kinases. Compared to the control calli, the phosphorylation level of CsABI5 was higher in calli overexpressing *CsSRK2A* or *CsCIPK6* (Figure 6A,D). In contrast, silencing *CsSRK2A* or *CsCIPK6* resulted in a decrease in the level of phosphorylated CsABI5 (Figure 6A,D). Correspondingly, transcript levels of *CsTST2* were found to be elevated by approximately 2–3‐fold with the overexpression of *CsSRK2A* or *CsCIPK6* compared to control lines. In contrast, silencing *CsSRK2A* or *CsCIPK6* led to a reduction in expression of *CsTST2* by approximately 50% (Figure 6B,E). These alterations in gene expression ultimately led to a corresponding increase or decrease in the accumulation of fructose, glucose and sucrose (Figure 6C,F). These results indicate that CsCIPK6 and CsSRK2A facilitate sugar accumulation through the phosphorylation of CsABI5, which in turn enhances the expression of *CsTST2*.

**FIGURE 7:**
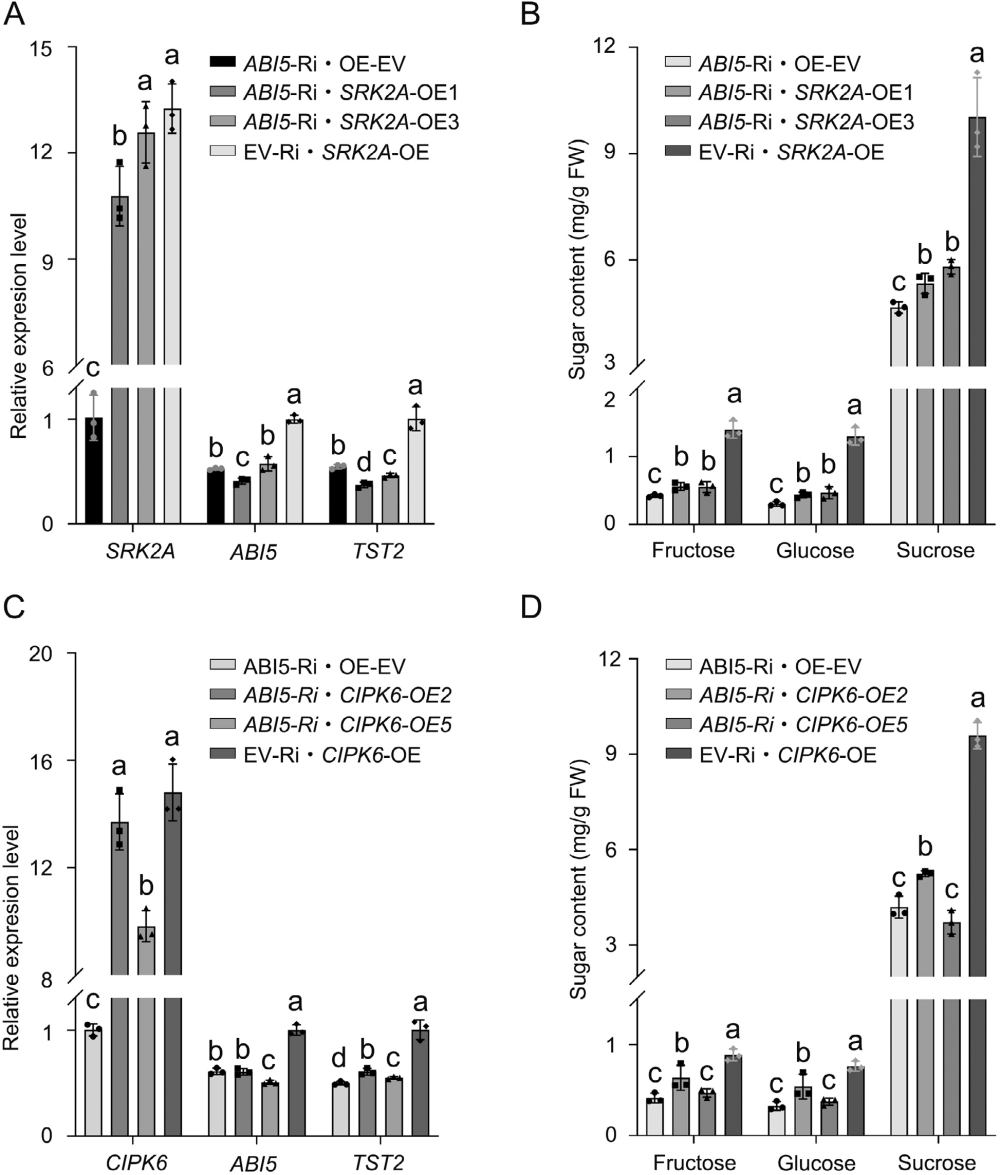
CsABI5 is indispensable for CsSRK2A or CsCIPK6 mediated upregulation of the *CsTST2* transcript level and sugar content. (A) In contrast to the overexpression of *CsSRK2A* in EV‐Ri transformed calli (EV‐Ri·*SRK2A*‐OE), stable overexpression of *CsSRK2A* in *CsABI5*‐silenced calli (*ABI5*‐Ri·*SRK2A*‐OE) behaved consistently with calli silenced with CsABI5 alone (*ABI5*‐Ri·EV‐OE), neither of which efficiently induced increased *CsTST2* expression. Expression of the corresponding gene in the control calli was set to ‘1’. (B) On the basis of *CsABI5* silencing (*ABI5*‐Ri·EV‐OE), an increase in Cs*SRK2A* expression (*ABI5*‐Ri·*SRK2A*‐OE) caused only a very slight increase in fructose, glucose and sucrose content, whereas overexpression of *CsSRK2A* alone (EV‐Ri·*SRK2A*‐OE) resulted in a much greater accumulation of soluble sugars. (C) Consistent with the silencing of *CsABI5* calli alone (*ABI5*‐Ri·EV‐OE), increasing *CsCIPK6* expression on the basis of *CsABI5* silencing (*ABI5*‐Ri·*CIPK6*‐OE) did not, or only marginally, increase the transcriptional level of *CsTST2*. In contrast, the overexpression of *CsCIPK6* in the background of EV‐Ri (EV‐Ri·*CIPK6*‐OE) effectively increased the transcript level of *CsTST2*. (D) On the basis of *CsABI5* silencing (*ABI5*‐Ri·EV‐OE), the overexpression of *CsCIPK6* (ABI5‐Ri·*CIPK6*‐OE) did not or only caused a slight increase in fructose, glucose and sucrose content, whereas overexpression of *CsCIPK6* alone (EV‐Ri·*CIPK6*‐OE) resulted in a much greater accumulation of soluble sugars. Transgenic calli samples were collected at 21‐day intervals, serving as independent biological replicates. Bars represent mean ± SD (*n* = 3 independent biological replicates). Different letters indicate significant differences as assessed by two‐tailed Student's *t*‐test (*p* < 0.05).

To examine the role of CsABI5 in this process, we subsequently overexpressed *CsSRK2A* and *CsCIPK6* in conjunction with *CsABI5*‐RNAi or EV‐RNAi, resulting in the establishment of stable *CsSRK2A*‐OE/*CsABI5*‐RNAi and *CsCIPK6*‐OE/*CsABI5*‐RNAi co‐transformed calli. The expression level of *CsTST2* and sugar levels in the *CsSRK2A*‐OE/*CsABI5*‐RNAi and *CsCIPK6*‐OE/*CsABI5*‐RNAi calli were similar to or only marginally higher than those in the *CsABI5*‐RNAi calli. However, the sugar levels were significantly lower than those observed in the EV‐RNAi/*CsSRK2A*‐OE and EV‐RNAi/*CsCIPK6*‐OE samples (Figure 7A–D). This indicates that the effect of CsCIPK6 and CsSRK2A on sugar accumulation is dependent on CsABI5. Collectively, these findings indicate that both CsSRK2A and CsCIPK6 play a positive role in regulating sugar accumulation by enhancing the phosphorylation level of CsABI5, which in turn promotes the transcription of *CsTST2*.

### 2.7 CsABI5 Binds to the Promoter of *CsCIPK6* and Promotes Its Transcription

**FIGURE 8:**
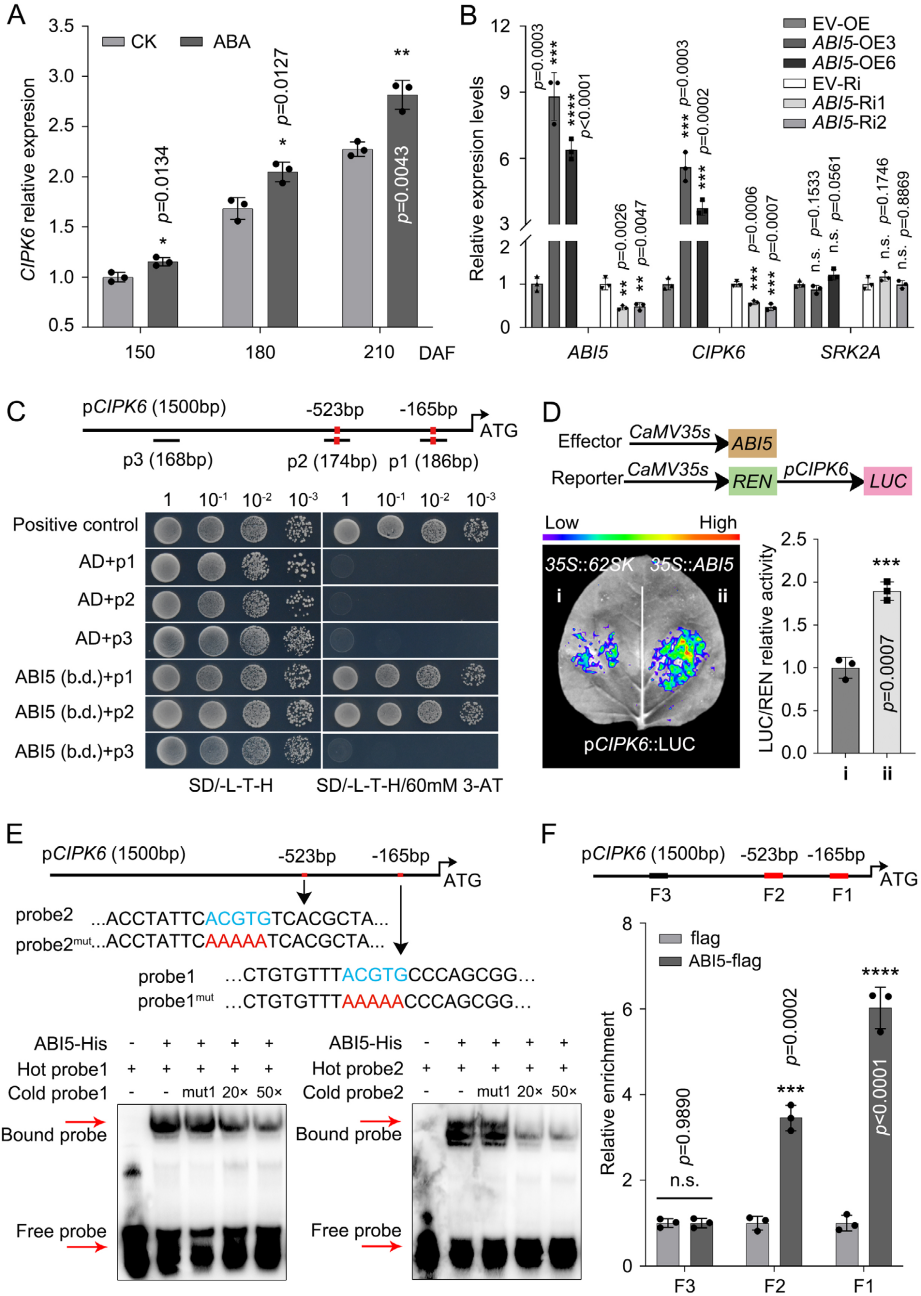
*CsCIPK6* expression is transcriptionally regulated by CsABI5. (A) The expression level of *CsCIPK6* in ABA‐treated and control fruit pulp was detected using RT‐qPCR. *CsActin* was used as an internal standard. The expression level of *CsCIPK6* in the 150 DAF untreated pulp was set to ‘1’. (B) Expression levels of *CsCIPK6* and *CsSRK2A* were examined in *CsABI5* transgenic calli. The transcript levels of the corresponding genes in empty vector‐transformed calli were set to ‘1’. (C) Y1H analysis showed that the DNA binding domain (b.d.) of CsABI5 binds to fragments p1 and p2 of *pCsCIPK6* with the ABRE element, but not fragment p3 without the ABRE element. Red rectangles represent ABRE elements. Each colony was dissolved in 50 μL of sterile water, subjected to a gradient dilution and cultured in SD/−L−T−H medium for 3 days. (D) In vivo imaging (left) and relative firefly luciferase activity assays (right) performed in *N. benthamiana* leaves showed that CsABI5 enhances the transcriptional activity of the *CsCIPK6* promoter (*pCsCIPK6*). Injection of the empty vector on one side served as a control. (E) EMSA analysis showed that CsABI5 binds to two ABRE elements in *pCsCIPK6* in vitro. Biotin‐free labelled probes were used as competitors (cold probe), and mutations in the ABRE elements caused the loss of competition of the cold probe. Red rectangles indicate DNA probes containing the ABRE element. (F) ChIP‐PCR assay demonstrated that CsABI5 binds to the promoter of *pCsCIPK6* in vivo. Chromatin immunoprecipitation was performed using samples with stable expression of CsABI5‐Flag or empty Flag control. ChIP‐PCR was conducted to assess the enrichment of F1, F2 containing ABRE elements and F3 lacking the ABRE element. The relative enrichment in empty Flag control calli was set as ‘1’; red rectangles represent ABRE elements. Bars represent mean ± SD (*n* = 3 independent biological replicates). Asterisks indicate significant differences by two‐tailed Student's *t*‐test (*p < 0.05, **p < 0.01, ***p < 0.001, ****p < 0.0001). n.s., not significant.

To determine if ABA signalling operates in conjunction with calcium signalling on sugar accumulation in citrus fruit, we investigated the relationship between these two signalling pathways. Gene expression analysis indicated that the transcript level of *CsCIPK6* increased progressively during fruit development, whereas *CsSRK2A* did not display a comparable pattern (Figure S13A). Moreover, *CsCIPK6* expression was indeed induced by ABA (Figure 8A). This observation prompted us to look into the expression dynamics of *CsCIPK6* and the potential regulatory role of CsABI5. The transcript‐level analyses of *CsABI5*, *CsCIPK6* and *CsSRK2A* during fruit development revealed a good correlation between the expression levels of *CsABI5* and *CsCIPK6*. In contrast, no such correlation was observed with *CsSRK2A* (Figure S13B). This finding was subsequently confirmed by the detection of gene expression levels of *CsCIPK6* and *CsSRK2A* in the *CsABI5* transgenic calli, which showed a positive correlation between the expression of *CsCIPK6* and *CsABI5*, with a simultaneous increase or decrease in transcript levels. In contrast, the expression of *CsSRK2A* exhibited only minor fluctuations within a limited range and remained unaffected by changes in *CsABI5* expression levels (Figure 8B). Subsequently, we analysed the promoter sequence of *CsCIPK6* and identified two characteristic ABRE elements (Figure S13C). These findings led us to hypothesise that CsABI5 may modulate the expression of *CsCIPK6*.

To confirm this hypothesis, we cloned fragments of the *CsCIPK6* promoter with (p1, p2) or without (p3) the ABRE element into the pHIS2 vector and then co‐transfected each of them into the yeast strain Y187 with either *CsABI5* (b.d.)‐pGADT7 or pGADT7. Positive colonies of *CsABI5* (b.d.)‐pGADT7 in combination with p1 or p2 grew normally on SD/−H/−L/−T medium containing 80 mM 3‐AT, whereas those containing the combination of pGADT7 or p3 fragments did not (Figure 8C). In addition, LUC assays demonstrated a significant increase in LUC activity when co‐expressing 35S::*CsABI5* and *pCsCIPK6*::LUC compared to 35S::62SK (Figure 8D). The subsequent EMSA experiments showed that the CsABI5‐His fusion protein exhibited binding affinity towards the *CsCIPK6* promoter DNA probe containing the ABRE element, whilst a cold probe with the ABRE element mutated lost its ability to compete with the biotin‐labelled probe for binding as the regular cold probe did (Figure 8E). The ChIP‐PCR assay with the 35S::*CsABI5*‐3 × Flag transgenic citrus calli found that the immunoprecipitation of CsABI5 resulted in a higher enrichment of two *CsCIPK6* promoter fragments containing ABRE elements, whereas no significant enrichment was obtained for fragments without the ABRE element (Figure 8F). The findings suggest that CsABI5 exerts a positive regulatory influence not only on *CsTST2* but also on *CsCIPK6*, thereby facilitating crosstalk and signalling between ABA and calcium for enhanced sugar accumulation in citrus fruit.

## 3 Discussion

Sugar accumulation in fleshy fruits is largely controlled by intracellular transport of sugars from the cytosol to the vacuole. Our earlier work showed that CsTST2 is primarily responsible for mediating sugar transport across the tonoplast in citrus fruit (Li et al. 2024; Mao et al. 2024). Here, we show that the application of exogenous ABA or calcium enhances the accumulation of soluble sugars in citrus fruit (Figure 1). CsABI5 transcriptionally activates *CsTST2* expression in response to ABA and calcium signals. Both CsSRK2A, a key player in ABA signalling, and CsCIPK6, a kinase linked to calcium signalling, phosphorylate CsABI5 at distinct amino acid sites to enhance its transcriptional activity. In addition, CsABI5 regulates the expression of *CsCIPK6* by binding to its promoter, thereby establishing a positive feedback loop between ABA and calcium signalling that leads to higher *CsTST2* expression and more sugar accumulation. These findings reveal a novel regulatory mechanism for ABA and calcium to co‐regulate sugar accumulation via ABI5 in a non‐climacteric fruit.

We first confirmed the association between the level of ABA and accumulation of soluble sugars during citrus fruit development reported earlier (Romero et al. 2012; Feng et al. 2021) (Figure 1B). To explore the molecular link between ABA and the expression of *CsTST2*, we identified several TFs that might regulate *CsTST2* transcription through co‐expression screening (Figure S1A and Table S1). The AREB TF CsABI5, a crucial component in the ABA signalling pathway, was amongst the identified TFs (Zhao et al. 2020; Peng et al. 2022). Considering the significant correlation between *CsABI5* and *CsTST2* at both the protein and transcript levels (Figure 2C,D and Figure S3), we performed Y1H, EMSA, ChIP‐PCR and LUC assays, which confirmed its specific binding to and transactivation effect on the promoter of *CsTST2* (Figures 2 and 3). Furthermore, overexpression and RNAi of *CsABI5* enhanced and decreased the expression level of *CsTST2*, respectively, leading to corresponding changes in the accumulation of soluble sugars in the juice sacs and calli of citrus fruit (Figure 3). These data provide compelling evidence that CsABI5 is a critical regulator for *CsTST2* expression that underlies sugar accumulation in citrus fruit.

To identify proteins that regulate the ABI5 TF in the ABA signalling pathway, we conducted a Y2H library screening and successfully identified the SnRK2 family member of CsSRK2A. We confirmed that CsSRK2A physically interacts with CsABI5 via both in vitro and in vivo assays (Figure 4). The phosphorylation of ABI5 by SnRK2s generally takes place at serine (Ser) or threonine (Thr) residues located within the R‐X‐X‐S/T or S/T‐X‐X‐E/D motifs (Furihata et al. 2006). We identified four distinct phosphorylation sites, Ser40, Ser109, Thr112 and Ser134 in the CsABI5 protein sequence by CsSRK2A based on the LC–MS/MS assay (Figure S7). Notably, the sequence surrounding Ser134 is IAGG‐S‐GVGG and diverges from previously documented phosphorylation recognition patterns, suggesting that CsSRK2A may play a role in the accumulation of soluble sugars in citrus fruit with a distinct phosphorylation mechanism. The phosphorylation modification of TFs enables kinases to regulate the expression of target genes by altering the regulatory activity of TFs (Yu et al. 2015). In our case, the phosphorylation of CsABI5 by CsSRK2A significantly enhances the transcriptional activity of *CsTST2* (Figure 5A–C). In contrast, the substitution of un‐phosphorylatable alanine (Ala) for Ser/Thr at positions 40, 109, 112 and 134 hinders the ability of CsSRK2A to phosphorylate CsABI5, consequently diminishing the transcriptional activity of *CsTST2* by CsABI5 (Figure 5D–F).

In addition to CsSRK2A, CsCIPK6 was also found to interact with CsABI5 in vitro and in vivo (Figure 4). CIPKs are generally recruited to diverse cellular locations by their cooperative chaperones, referred to as CBLs, to respond to calcium signals and achieve functional diversity (Luan and Wang 2021; Sun, Xia, Deng, et al. 2024). By doing a comprehensive screening and validation of the complete set of CsCBL members in sweet orange, we identified cytoplasmic and nuclear‐localised CsCBL1 as the reciprocal interacting protein of CsCIPK6 (Figures S6 and S7). Previous studies indicate that the distinct N‐terminal regions of CBL proteins play a significant role in determining their specific cellular targeting destinations. In particular, the N‐myristoylated sequence M‐G‐X‐X‐X‐(S/T) of AtCBL1 was identified as a potential marker for the localization of CBL proteins at the cellular membrane (Batistic et al. 2008). However, subsequent investigations demonstrated that fluorescent signals produced by GFP‐tagged AtCBL4 and AtCBL5 fusion proteins were detected in multiple cellular compartments, including the cytoplasm and nucleus, even in the presence of this sequence (Batistic et al. 2010). To investigate the impact of the 12 amino acids containing the M‐G‐X‐X‐X‐(S/T) sequence on the localization of CsCBL1, we substituted the amino acids at positions 5 (Q), 8 (V) and 11 (Q) in CsCBL1 with 5 (H), 8 (A) and 11 (E) from *Arabidopsis*. Interestingly, this substitution did not affect the cytoplasmic and nuclear localization of CsCBL1 (Figure S7). The result indicates that whilst the N‐terminal structural domain of CBL proteins plays a significant role in their localization, it is not the sole determinant for the targeting mechanism of CBL proteins. This observation supports the view that CsCBL1 is involved in decoding calcium signalling and facilitating the interaction between CsCIPK6 and CsABI5 within the nucleus. Similarly, the subcellular localization characteristics of CsSRK2A are also consistent with those of CsCIPK6 (Figure S5). The interaction between CsCIPK6 and CsABI5, akin to the mechanism of action of CsSRK2A, led to the phosphorylation of Ser39 and Ser40 residues in CsABI5, subsequently enhancing the transcriptional activity of CsABI5 towards *CsTST2* (Figure 5G–I and Figure S9).

**FIGURE 9:**
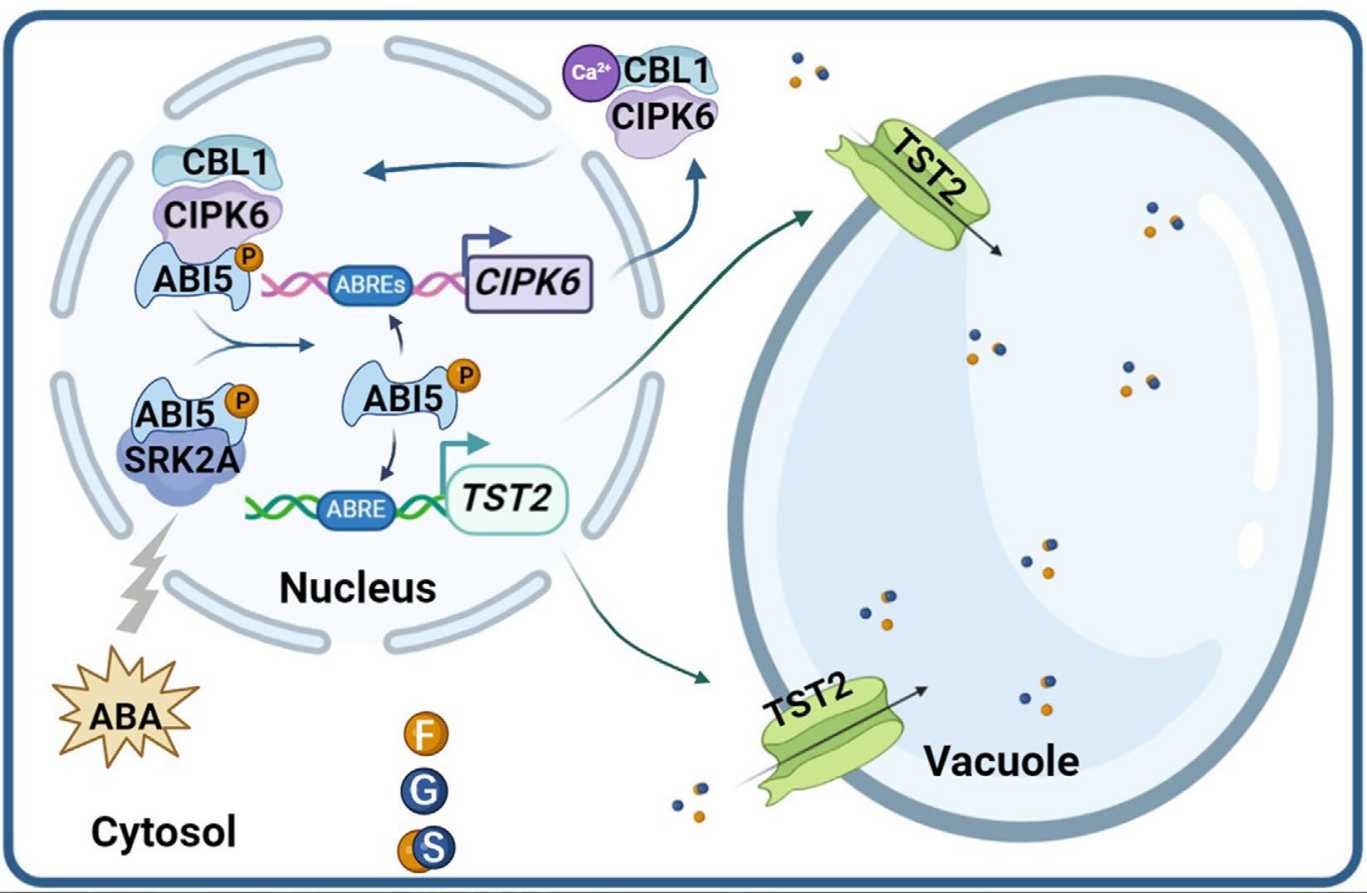
A model of co‐regulation of soluble sugar accumulation in citrus fruit by ABA and calcium signalling via the CsSRK2A/CsCIPK6‐CsABI5‐*CsTST2* module. *CsTST2* encodes a sugar transporter localised in the tonoplast that mediates the transport of sugars into the vacuole in sweet orange. The expression of *CsTST2* is regulated by the transcription factor CsABI5. CsSRK2A and CsCBL1/CIPK6 phosphorylate different amino acid sites in CsABI5 to enhance its transcriptional activation of *CsTST2*. CsABI5, in return, activates the expression of *CsCIPK6* to form a positive feedback loop, which enables the sweet orange fruit to accumulate high levels of soluble sugars during development.

The interplay between calcium and ABA signalling has been implicated in plant abiotic stress responses (Kim et al. 2003; Kudla et al. 2018). In *Arabidopsis*, CNGC5/6/9/12 were identified as ABA‐activated plasma membrane Ca^2+^ channels, where loss‐of‐function mutations result in impaired stomatal closure under drought conditions due to defective ABA signalling perception (Tan et al. 2023). In *Oryza sativa* , the CDPK OsDMI3 mediates activation of the MAPK cascade through direct phosphorylation of OsMKK1, thereby enhancing ABA signalling‐induced antioxidant defence mechanisms and stomatal closure responses (Chen et al. 2021). During citrus fruit development, the contents of ABA and calcium were continuously increased, accompanied by the accumulation of high levels of sugars and secondary metabolites in the fruit flesh (Romero et al. 2012; Khan et al. 2023; Zhang et al. 2023; Sun, He, Feng, et al. 2024; Zhang, Xu, et al. 2024). However, the molecular connection between these calcium and ABA signalling pathways has not been demonstrated in fruit quality formation. Our data clearly indicate that both ABA and calcium enhance sugar accumulation in citrus fruit (Figure 1). In this process, CsABI5 serves as a novel convergence point for calcium and ABA signalling pathways in regulating sugar accumulation in citrus. In citrus calli, overexpression of *CsCIPK6* or *CsSRK2A*, in conjunction with the silencing of *CsABI5*, did not effectively enhance the transcript levels of *CsTST2* or elevate sugar levels (Figure 7), indicating that CsABI5 is indispensable for the integration of ABA and calcium signalling during sugar accumulation in citrus. This is consistent with previous findings that SnRK2s and CIPKs can share overlapping substrates (Steinhorst and Kudla 2014; Edel and Kudla 2016; Kudla et al. 2018). Although both CsCIPK6 and CsSRK2A phosphorylate CsABI5, it is noteworthy that CsABI5 has two sites phosphorylated by CsCIPK6 (Figure S9), vs. four sites by CsSRK2A (Figure S8). This suggests that CsSRK2A may possess a greater capacity to phosphorylate CsABI5 compared to CsCIPK6. Moreover, during fruit development, the expression of *CsCIPK6* was characterised by a continuous increase concomitant with soluble sugar accumulation, different from the stable expression pattern of *CsSRK2A* (Figure S11A). This distinct expression pattern may partially compensate for the fewer sites in CsABI5 phosphorylated by CsCIPK6. It was suggested earlier that calcium signalling may play a more flexible regulatory role in the long‐term response to ABA signal in an integrated manner (Edel and Kudla 2016; Kudla et al. 2018). Our initial analysis of the promoter region of *CsCIPK6* revealed the presence of two putative distinct ABA response elements (ABRE) (Figure S11C). Subsequent analyses demonstrated a good correlation between the expression levels of *CsABI5* and *CsCIPK6* during fruit development, which was further corroborated by the transcript level of *CsCIPK6* in *CsABI5* transgenic calli (Figure 8B). Finally, we demonstrated that CsABI5 binds to the two ABRE elements within the *CsCIPK6* promoter, thereby activating its expression through Y1H and Luc assays, EMSAs and ChIP‐PCR analyses (Figure 9). This activation pattern resembles that of *CsTST2* and explains the consistent upregulation of *CsCIPK6* expression during citrus fruit development.

Collectively, the data presented in this work show that CsSRK2A, a kinase involved in the ABA signalling pathway, and CsCBL1/CIPK6, a calcium sensor/kinase complex associated with the calcium signalling pathway, promote the expression of the tonoplast sugar transporter gene *CsTST2* by phosphorylating its transcriptional activator CsABI5. In addition, CsABI5 exerts a positive feedback activation on the expression of *CsCIPK6*, thereby regulating calcium signalling over an extended period to sustain its stimulation of *CsTST2* expression that enhances the accumulation of soluble sugars in the citrus vacuole (Figure 9). This novel CsSRK2A/CsCIPK6‐CsABI5‐CsTST2 module underlies sugar accumulation in citrus fruit, revealing the intricate coordination between ABA and calcium signalling in regulating the quality of citrus, a non‐climacteric fleshy fruit, which may serve as a model for understanding sugar accumulation in other fleshy fruits.

## 4 Materials and Methods

### 4.1 Plant Materials, Growth Conditions and Treatments

Navel orange ( *Citrus sinensis* cv. Qingjia; *Citrus sinensis* cv. Fukumoto) and pumelo fruits ( *Citrus grandis* Osbeck ‘Hirado Buntan’, HB) were harvested from trees grown at the Citrus Germplasm Resource Center of Huazhong Agricultural University (Wuhan, China). These trees were maintained following standard horticultural management practices. Qingjia and Fukumoto oranges were harvested at 30‐day intervals from 150 DAF (days after flowering) to 210 DAF. The juice sacs near the equatorial plane of the fruits were quickly peeled off, frozen in liquid nitrogen and stored at −80°C for the detection of calcium, ABA, soluble sugars and gene expression. Nine fruits from three healthy trees were used as independent biological replicates. On‐tree fruit treatments were conducted every 7 days, commencing at 120 DAF. Briefly, whole fruits were submerged in an aqueous solution of 100 μM ABA (Macklin, Cat. A800056, Shanghai, China) containing 0.1% Tween 20 for 5 min. For the calcium treatment, 100 mM of Ca (NO_3_)_2_ · 4H_2_O (Sigma‐Aldrich, Cat. 237124, USA) were sprayed uniformly on the surface of the fruits. Fruits sprayed or submerged in water or solution buffer were used as controls. HB pumelo fruits were harvested at 180 DAF, and their fresh juice sacs were stripped for *Agrobacterium*‐mediated transfection. The transfected juice sacs were stored at −80°C after rapid freezing in liquid nitrogen for measuring soluble sugars and gene expression. Wide type (WT) calli induced from reticulata (*Citrus unshiu* Marc. cv. Guoqing No. 1) were cultured on MT medium without hormones. The medium used to culture the transformed overexpression and RNAi calli was additionally supplemented with 40 mg/L of Hygromycin B and Kanamycin Monosulfate, respectively. The medium used to culture overexpression and RNAi co‐transformed calli was supplemented with both of the above antibiotics. All calli were grown at 24°C under dark conditions and sub‐cultured every 3 weeks. Tobacco plants (*N. benthamiana*) were grown in a mixture of grass charcoal, vermiculite and perlite (3:2:1, v/v/v) under 24°C in a light/dark (14 h/10 h) cycle.

### 4.2 Measurement of Soluble Sugars, Calcium and ABA

Soluble sugars were extracted using 75% (v/v) methanol as previously described (Li, Meng, et al. 2020), with ribitol (0.12 mg every sample) added as an internal standard. After removal of non‐polar metabolites with chloroform, 200 μL of each aqueous phase sample was dried under vacuum without heat and then derivatized with methoxyamine hydrochloride and N‐methyl‐N‐trimethylsilyl‐trifluoroacetamide sequentially. The Agilent 6890B Gas Chromatography System (Agilent Technologies, Palo Alto, CA, USA) equipped with a flame ionisation detector and a non‐polar HP‐5 (5%‐phenyl)‐methylpolysiloxane column (30.0 m × 0.32 mm × 0.25 μm) was used to analyse the sugars and organic acids. Three independent extractions were performed for each sample. For the determination of ABA, 0.5 g of juice sacs was ground in liquid nitrogen and subsequently mixed with 1.5 mL of 80% methanol containing 1 mM of 2,6‐di‐tert‐butyl‐4‐methylphenol (BHT). Following a 20‐min ultrasonication process, the mixture was extracted for 12 h at −20°C. The collected supernatant was thoroughly evaporated and then redissolved in 700 μL of 0.4 M phosphate buffer (pH 8.0). After the removal of pigments using petroleum ether, 20 μL of 2 M NaOH and 80 mg of insoluble polyvinylpyrrolidone (PVP) were added to the inorganic phase. The supernatant was then collected via centrifugation, and 35 μL of formic acid was added. The sample was extracted three times with 700 μL of ethyl acetate. The organic phases were combined, concentrated to dryness and redissolved in 0.5 mL of methanol for analysis. Following the method previously described (Zhai et al. 2023), the isocratic dilution of the sample was conducted at 40°C with an injection volume of 10 μL using the 20ADXR‐8040 HPLC‐MS/MS system (Shimadzu, Japan) loaded with a Shim‐pack GIST‐C18 column (2 μm × 2.1 mm × 50 mm; Shimadzu, Kyoto, Japan). For measuring calcium, the samples were digested with HNO_3_‐HClO_4_ (v/v, 4:1) as previously described (Zhou et al. 2018). Subsequently, the concentration of calcium in the digested solution was quantified utilising Inductively Coupled Plasma‐Optical Emission Spectrometer (Agilent, 5110VDV ICP‐OES, USA). Fruit Ca concentrations were expressed on a dry weight basis.

### 4.3 RNA Extraction and RT‐qPCR

RNA extractions and cDNA synthesis were performed as previously described (Mao et al. 2024). Quantification of gene mRNA levels was conducted with the QuantStudio 7 Flex Real‐Time PCR system (Applied Biosystems, Singapore), with *CsActin* as a reference. Relative expression values were calculated using the 2^−ΔΔCt^ method. Fold change and SD were calculated from three biological replicates. All related primers are listed in Table S3.

### 4.4 *Agrobacterium*‐Mediated Transformation of Citrus Calli and Juice Sacs

For recombinant vector construction, the CDS sequences of *CsABI5*, *CsCIPK6* and *CsSRK2A* were cloned into the pK7WG2D or pH 7WG2D vectors to generate overexpression constructs, whilst portions of the sequences were inserted into the pK7GWIWG2D(II) vector for RNAi of specific genes. Primers used for vector construction are listed in Table S3. To obtain stably transformed calli, the sub‐cultured calli were immersed in a resuspension of *Agrobacterium tumefaciens* EHA105 containing either the pH7WG2D or pK7GWIWG2D(II) recombinant vectors and vacuumed for 5 min at 0.8 MPa. Subsequently, positive calli were screened on MT medium containing appropriate antibiotics. For transformation, *A. tumefaciens* EHA105 containing the pK7WG2D or pK7GWIWG2D(II) recombinant vector was used for the overexpression or silencing of specific genes in citrus juice sacs. The transcript levels of the target genes were detected using RT‐qPCR described above using primers listed in Table S3.

### 4.5 Subcellular Localization Analysis

The CDSs of *CsABI5*, *CsCIPK6*, *CsCBL1*, *CsCBL1*(mut) and *CsSRK2A* were introduced into the pRI101 vector containing the YFP‐tag, respectively, and then transformed into *Agrobacterium* GV3101 as previously described (Mao et al. 2024). AtH2B fused with mCherry was used for co‐localization analysis. Positive *Agrobacterium* colonies were injected into *N. benthamiana* leaves to express the fusion proteins for 3 days for detection of YFP and mCherry signals on a laser scanning confocal microscope (Leica TCS‐SP8, Germany) in multichannel mode. The excitation wavelengths of YFP and mCherry were 514 nm and 587 nm, respectively, and the detection wavelengths of their emission signals were 520–540 and 600–620 nm, respectively. Primers used for gene cloning are listed in Table S3.

### 4.6 Transcription Factor–Promoter Interaction Assays

#### 4.6.1 Y1H Assay

The sequence of the DNA‐binding domain (BD) of *CsABI5* was ligated into the pGADT7 vector, and promoter fragments of *CsTST2* and *CsCIPK6* with or without ABRE elements were ligated into the pHIS2 vector. These pGADT7 and pHIS2 vector pairs were transformed into the Y187 yeast strain and cultured on SD/−T/−H/−L medium, and positive clones were transferred to SD/−T/−H/−L medium containing 40/60/70 mM 3‐AT medium to assess protein binding to DNA. The primers used are listed in Table S3.

#### 4.6.2 Dual Luciferase (LUC) Activity Assay

The promoters of *CsTST2* and *CsCIPK6* were inserted into the reporter vector pGreenII 0800‐LUC, respectively, whilst CDSs of *CsABI5*, *CsABI5* ^2m^ and *CsABI5* ^4m^ were integrated into the vector pK7WG2D, which contains 3 × Flag. The CDSs of *CsCIPK6* and *CsSRK2A* were inserted into the vector pGWB417, which is tagged with 4 × MYC. Agrobacterium strain GV3101 pairs containing the appropriate vectors were used to infiltrate *N. benthamiana* leaves. After 3 days, the relative activity of the LUC enzyme was determined using a Spark Multifunctional microplate reader (TECAN, Spark Austrian) following the manufacturer's instructions (Yeasen Biotechnology, Cat. 11402ES60, Shanghai, China). The LUC signal intensity was measured using Plant Live Imaging System (Berthold Technologies, LB 985, Germany). Associated proteins were detected using Anti‐DDDDK‐Tag Rabbit mAb (ABclonal Technology, Cat. AE063, Wuhan, China), MYC‐Tag Rabbit mAb (ABclonal Technology, Cat. AE070, Wuhan, China), GFP‐Tag Rabbit mAb (ABclonal Technology, Cat. AE078, Wuhan, China) and HRP‐conjugated Goat anti‐Rabbit IgG (ABclonal Technology, Cat. AS014, Wuhan, China), all at a dilution ratio of 1:10 000. The above experiments were performed in three biological replicates. Primers are listed in Table S3.

#### 4.6.3 Electrophoretic Mobility Shift Assay (EMSA)

The CsABI5‐His fusion protein was expressed in *Escherichia coli* strain BL21 (DE3). The *CsTST2* and *CsCIPK6* promoter fragments containing the ABRE elements (ACGTG) were labelled with biotin and synthesised by Sangon Company (Shanghai, China). The unlabelled probe with the same sequence or mutant ABRE element (AAAAAA) was used as a competitor. The CsABI5‐His protein was mixed with the probes and combined following the instructions of the Chemiluminescent EMSA Kit (Beyotime, Cat. GS009, Shanghai, China). The mixtures were incubated at 24°C for 30 min and separated by 6% non‐denaturing PAGE gels, and signals were detected by chemiluminescence imaging (Tanon 5200, Shanghai, China). The sequence of primers and probes are listed in Table S3.

#### 4.6.4 ChIP‐PCR Assay

The coding sequence of *CsABI5* without the stop codon was inserted into the pH 7WG2D vector containing a C‐terminal 3 × Flag. This 35S‐driven overexpression structure was transformed into citrus calli for ChIP‐qPCR analysis, with the citrus calli harbouring the empty vector of 35S:: 3 × Flag as a control. Anti‐C‐Flag Nanobody Magarose Beads (AlpaLifeBio, Cat. KTSM1338, Shenzhen, China) were used to immunoprecipitate protein‐DNA complexes for further analysis of the binding of CsABI5 to target DNA in vivo as previously described (Dahro et al. 2022). Primers of *CsTST2* and *CsCIPK6* promoters for ChIP‐PCR are listed in Table S3.

### 4.7 Protein–Protein Interaction Assays

#### 4.7.1 Y2H Assay

The cDNA libraries were constructed by Oebiotech (Shanghai, China), and the mRNA was extracted from ABA‐ and calcium‐treated sweet orange juice sacs, respectively. The *CsABI5* de‐activated CDS sequence (1–1287 bp, 1‐429aa) was ligated into the pGBKT7 vector carrying the sequence encoding the GAL4 DNA binding domain (BD) as bait for library screening. The successfully transformed positive clones were subsequently identified by PCR and sequenced. To detect protein–protein interactions, the coding sequence of *CsCIPK6* was constructed into the pGADT7 and pGBKT7 vectors carrying sequences encoding the GAL4 activation domain (AD) and the GAL4 DNA binding domain (BD), respectively, and the full‐length CDS sequences of *CsCBL1* and *CsSRK2A* were cloned into pGADT7 vectors according to the manufacturer's instruction (Takara & Clontech, Cat. 630489, Japan). The AD and BD plasmid pairs were transformed into Y2H‐gold yeast strain and cultured on SD/−L/−T medium for 3–44 days. The mono‐clones were transferred to SD/−A/−H/−L/−T medium for 3–44 days to assess protein interactions. Primers are listed in Table S3.

#### 4.7.2 Bimolecular Fluorescence Complementation (BiFC) Assay

For the BiFC assay, the coding sequence of *CsSRK2A* or *CsCIPK6* was inserted into the pSYPNE vector containing the N‐terminal YFP, whereas that of *CsABI5* was introduced into the pSYPCE vector containing the C‐terminal YFP. The *AtH2B*‐mCherry serves as a cell nucleus marker. The recombinant vectors were individually transformed into *Agrobacterium* strain GV3101 and then co‐injected into the leaves of *N. benthamiana*. Fluorescence signals were detected after 3 days using a laser confocal microscope (Leica TCS‐SP8, Germany). The excitation wavelength for YFP was set at 514 nm, with the emission wavelength range recorded between530 and 5500 nm. For mCherry, the excitation wavelength was 587 nm, and the corresponding emission wavelength range was between 600 and 6200 nm. The primers are listed in Table S3.

#### 4.7.3 Pull‐Down Assay

The CDS of *CsABI5* was cloned into the pET32a (+) vector containing a His‐Tag, whereas the CDS of *CsCIPK6* and *CsSRK2A* were ligated into pGEX4T‐1 containing a GST‐Tag, respectively. The recombinant plasmids were transformed into *E. coli* (BL21, DE3), and the fusion proteins were expressed in Luria‐Bertani broth containing 0.2 mM isopropyl‐beta‐D‐thiogalactopyranoside (IPTG) under 22°C for 10 h. For protein binding in vitro, CsCIPK6‐GST, CsSnRK2.1‐GST or GST was captured in binding buffer (160 mM NaCl, 10% Glycerol, 10 mM Na_2_HPO_4_, 2 mM KH_2_PO_4_) by BeyoGold GST‐tag Purification Resin (Beyotime, Cat. P2251, Shanghai, China), followed by incubation with the CsABI5‐His fusion protein at 4°C for 3 h. After sufficient washing with binding buffer to remove non‐specific binding proteins, the prey protein was eluted with elution buffer (50 mM Tris–HCl, pH 7.8) supplemented with 10 mM glutathione (GSH). The elution products were used for Western Blot analyses involving His (ABclonal Technology, Cat. AE003, Wuhan, China), GST (ABclonal Technology Cat. AE001, Wuhan, China) or HRP‐tagged goat mouse antibodies (ABclonal Technology Cat. AS003, Wuhan, China), all at a dilution of 1:10 000, to assess the interaction of the two proteins in vitro. The primers are listed in Table S3.

#### 4.7.4 Luciferase Complementation Imaging (LCI) Experiment

The CDSs of *CsCIPK6* and *CsSRK2A* were ligated to the JW‐771‐nLUC vector, and those of *CsABI5* and *CsCBL1* were ligated to JW‐772‐cLUC, respectively, as previously described (Yue et al. 2023). Then, the constructs were individually introduced into *Agrobacterium* strain GV3101 and co‐expressed in *N. benthamiana* leaves. The chemiluminescence produced by LUC was observed after 3 days using the Plant Live Imaging System LB985 (Berthold Technologies, Germany). The primers are listed in Table S3.

#### 4.7.5 Co‐IP Assay

The coding sequence of *CsABI5* was cloned into the pK7WG2D vector containing a 3 × Flag tag, and the coding sequences of *CsCIPK6* and *CsSRK2A* were cloned into the pGWB417 vector containing a 4 × MYC tag, with the empty pGWB417 vector as a negative control. The recombinant vectors were then transformed into *Agrobacterium* strain GV3101 and co‐injected into the leaves of 4‐week‐old *N. benthamiana*. After 3 days, total proteins of *N. benthamiana* leaves were extracted using a lysis buffer containing 50 mM Tris–HCl (pH 7.5), 150 mM NaCl, 1 mM EDTA, 1% Triton‐100 and 1× protease inhibitor (Beyotime, Cat. P1045, Shanghai, China). Subsequently, MYC or CsCIPK6‐MYC, CsSRK2A‐MYC fusion proteins were co‐immunoprecipitated using Anti‐C‐Flag Nanobody Magarose Beads (AlpaLifeBio, Cat. KTSM1338, Shenzhen, China). Anti‐Flag, ‐MYC and HRP‐conjugated Goat anti‐Rabbit antibodies were the same as those described in the LUC assay at a dilution of 1:5000 for immunoblot analysis. Primers used are listed in Table S3.

### 4.8 Western Blotting Assay

Total proteins were extracted from citrus juice sacs, calli or *N. benthamiana* leaves. The protein concentrations were quantified using a protein assay kit (Beyotime, Cat. P0006, Shanghai, China) and subsequently adjusted to a uniform protein level across all samples. Specific monoclonal antibodies, Anti‐CsABI5 (targeting the specific peptide MFASDTYLMGSNINFKNFSNEPPNDVGKPP) and Anti‐CsTST2 (targeting the specific peptide IEKDMVPPAHGTLSSMRHGSQVQGNAGEPVGM) produced in rabbits (ABmart company, Shanghai, China) were used at a dilution of 1:3000. Tubulin bands were identified using an anti‐tubulin antibody (ABmart product No. M20005S) at a dilution of 1:10 000. Signals were detected by chemiluminescence imaging (Tanon 5200, Shanghai, China), and the quantification of the protein bands was performed using Image J software (version 1.4.3.67).

### 4.9 In Vitro and In Vivo Phosphorylation Assay

For in vitro phosphorylation assay, CDSs of *CsABI5* and *CsCBL1* were ligated into pET‐30a (+) and pET‐32a (+) vectors, respectively, for the production of the fusion proteins in *E. coli* (BL21, DE3). The CsCIPK6 and CsSRK2A fusion proteins carrying a GST‐tag were produced by expression of the pGEX4T‐1 vector. CsCIPK6‐GST (2 μg) and CsCBL1‐His (1.5 μg) were first incubated in reaction buffer (25 mM Tris–HCl, pH 7.5, 10 mM MgCl_2_, 1 mM CaCl_2_, 1 mM DTT, 2 mM ATP) for 1 h to activate CsCIPK6‐GST, followed by the introduction of the substrate proteins CsABI5‐His (10 μg) or CsABI5‐2A‐His (10 μg) for the phosphorylation reaction. In contrast, the phosphorylation of CsABI5‐His (10 μg) or CsABI5‐4A‐His (10 μg) by CsSRK2A‐GST (2 μg) was carried out in a reaction buffer without CaCl_2_. λ‐PPase (Beyotime, Cat. P2316S, Shanghai, China) was used to eliminate the phosphoryl groups on the substrate proteins. The reacted protein products were separated by 12% SDS‐PAGE gels and transferred to PVDF membranes. In vivo phosphorylation assays were conducted by extracting total proteins from transgenic calli or fruit flesh samples at different developmental stages using a lysis buffer that contained a combination of protease and phosphatase inhibitors (Beyotime, Cat. P1048, Shanghai, China), supplemented with an additional 30 μg/mL of an anti‐CsABI5 specific antibody. CsABI5 was subsequently immunoprecipitated using Protein A + G magnetic beads (Beyotime, Cat. P2108, Shanghai, China), following the manufacturer's guidelines. Aliquots of the samples were then separated using an 8% SDS‐PAGE gel for further analysis. The detection of phosphorylated bands is conducted using an anti‐pSer/pThr antibody (ECM Biosciences, PM3801, Versailles, KY, USA) at a dilution of 1:5000. The detection of phosphorylated sites on CsABI5 was performed by Bioprofile Technology Company (Shanghai, China). All primers are listed in Table S3.

### 4.10 Statistical Analysis

The experiments involved in this study were replicated three times. Results are expressed as mean ± SD. Student's *t*‐test was used to determine any statistical difference between two groups of data. Data were analysed using GraphPrism 10.2.3, Microsoft Excel 2019 and Sigma plot 14.0.

### 4.11 Accession Numbers

Sequence data from this study can be found at NCBI GenBank (https://www.ncbi.nlm.nih.gov/) or Citrus Pan‐genome to Breeding Database (http://citrus.hzau.edu.cn/index.php) under the accession numbers *CsDREB1B* (LOC102627433), *CsbHLH5* (LOC102610603), *CsVIP1* (LOC102630324), *CsERF1* (LOC102629603), *CsABI5* (LOC102608792), *CsTST2* (LOC102607273), *CsCIPK6* (LOC102625147), *CsSRK2A* (LOC102609931), *CsCBL1* (LOC102622664), *CsCBL2* (LOC102622316), *CsCBL3* (LOC102611616), *CsCBL4* (LOC102625423), *CsCBL5* (LOC107174223), *CsCBL6* (LOC102613753), *CsCBL7* (LOC102613852), *CsCBL8* (LOC102629470), *CsCBL9* (LOC102615552), *CsCBL10* (LOC102629757), *CsCBL11* (LOC102625529), *CsCBL13* (LOC102628611), *CsCBL14* (LOC18053860), *CsCBL15* (LOC102609927), *CsCBL16* (LOC102624742) and *CsActin* (XM_006486038).

## Author Contributions

C.L., J.‐H.L. and L.C. conceived and designed the study. Z.M. conducted the experiments and data analysis with the help from M.L., S.G., Z.Z. and X.Z. C.L. and Z.M. wrote the manuscript with inputs from all other authors. C.L. is responsible for the distribution of materials integral to the findings presented in this article.

## Conflicts of Interest

The authors declare no conflicts of interest.

## Supporting information


**Figure S1:** The screen of *CsTST2* co‐expression transcription factor and *CsTST2* promoter *cis*‐acting element analysis.
**Figure S2:** The subcellular localization of CsABI5 and schematic representation of the protein domain.
**Figure S3:** Detection and correlation analysis of *CsABI5* and *CsTST2* expression during citrus fruit development stages.
**Figure S4:** CsTST2 is indispensable for CsABI5‐mediated accumulation of soluble sugars.
**Figure S5:** Self‐activation detection and elimination of the CsABI5‐BD structure for a yeast two‐hybrid assay.
**Figure S6:** The subcellular localization of CsSRK2A and CsCIPK6.
**Figure S7:** The screen and further validation of CsCBL members that interact with CsCIPK6.
**Figure S8:** The subcellular localization of CsCBL1.
**Figure S9:** Detection of the phosphorylation level of CsABI5 during fruit development.
**Figure S10:** Amino acid residues of CsABI5 phosphorylated by CsSRK2A were analysed by LC–MS/MS.
**Figure S11:** Amino acid residues of CsABI5 phosphorylated by CsCIPK6 were analysed by LC–MS/MS.
**Figure S12:** Effect of overexpression of *CsSRK2A* or *CsCIPK6* in citrus juice sacs on *CgTST2* expression and sugar content.
**Figure S13:** Correlation analysis of *CsABI5* expression with *CsSRK2A* and *CsCIPK6* and the analysis of *cis*‐acting elements in the *CsCIPK6* promoter.
**Table S1:** The 113 genes co‐expressed with *CsTST2* in the STEM co‐expression analysis module.
**Table S2:** Potential interaction proteins with CsABI5 through yeast two‐hybrid screening.
**Table S3:** List of primers used in this study.

## Data Availability

All data are available within the manuscript and Supporting Information.
