# Supplementary material for: Abscisic Acid and Calcium Signals Convergently Regulate Sugar Accumulation by Orchestrating the SRK2A/CIPK6‐ABI5‐TST2 Module in Citrus
Source: Plant Biotechnol J. 2025 Sep 9;24(2):402–23. doi: 10.1111/pbi.70341 (PMC12906828; doi:10.1111/pbi.70341)
Supplement: Supplementary file 1 — Figure S1: The screen of CsTST2 co‐expression transcription factor and CsTST2 promoter cis‐acting element analysis. Figure S2: The subcellular localization of CsABI5 and schematic representation of the protein domain. Figure S3: Detection and correlation analysis of CsABI5 and CsTST2 expression during citrus fruit development stages. Figure S4: CsTST2 is indispensable for CsABI5‐mediated accumulation of soluble sugars. Figure S5: Self‐activation detection and elimination of the CsABI5‐BD structure for a yeast two‐hybrid assay. Figure S6: The subcellular localization of CsSRK2A and CsCIPK6. Figure S7: The screen and further validation of CsCBL members that interact with CsCIPK6. Figure S8: The subcellular localization of CsCBL1. Figure S9: Detection of the phosphorylation level of CsABI5 during fruit development. Figure S10: Amino acid residues of CsABI5 phosphorylated by CsSRK2A were analysed by LC–MS/MS. Figure S11: Amino acid residues of CsABI5 phosphorylated by CsCIPK6 were analysed by LC–MS/MS. Figure S12: Effect of overexpression of CsSRK2A or CsCIPK6 in citrus juice sacs on CgTST2 expression and sugar content. Figure S13: Correlation analysis of CsABI5 expression with CsSRK2A and CsCIPK6 and the analysis of cis‐acting elements in the CsCIPK6 promoter. Table S1: The 113 genes co‐expressed with CsTST2 in the STEM co‐expression analysis module. Table S2: Potential interaction proteins with CsABI5 through yeast two‐hybrid screening. Table S3: List of primers used in this study. [file PBI-24-402-s001.docx]

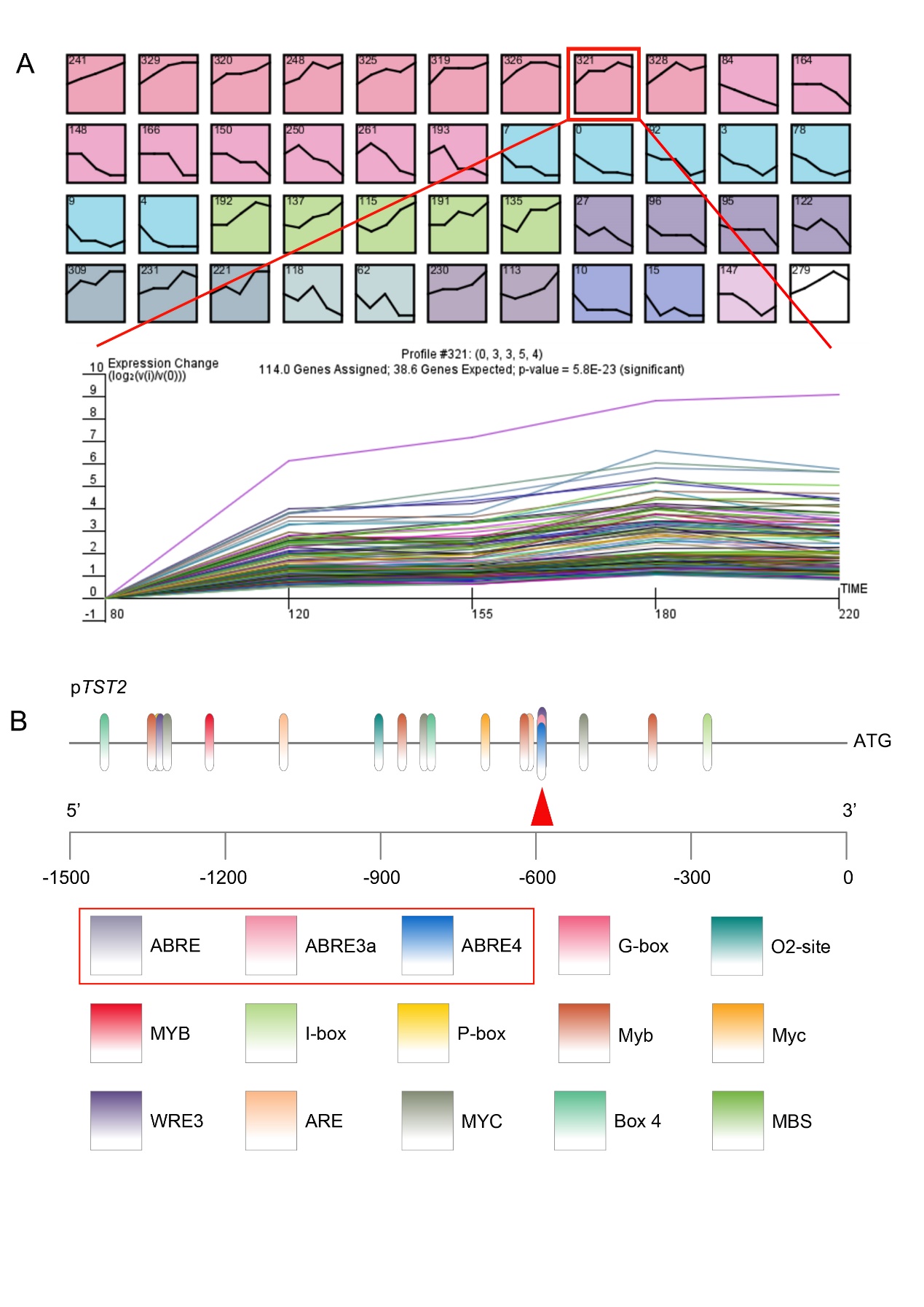


**Figure S1. The screen of *CsTST2* co-expression transcription factor and *CsTST2* promoter *cis*-acting element analysis.**

**(A)** A total of 114 candidate members were clustered in the target module by Short Time-series Expression Miner (STEM) cluster analysis to identify genes that are co-expressed with *CsTST2* during the citrus fruit development period (*P* Value = 5.8e^-23^). Maximum Unit Change in Model Profiles between Time Points was set to 330, and aximum Number of Model Profiles was set to 30. **(B)** The promoter of *CsTST2* (*pTST2*) was predicted to contain classical ABRE elements through the Plant CARE online platform (https://bioinformatics.psb.ugent.be/webtools/plantcare/html/).


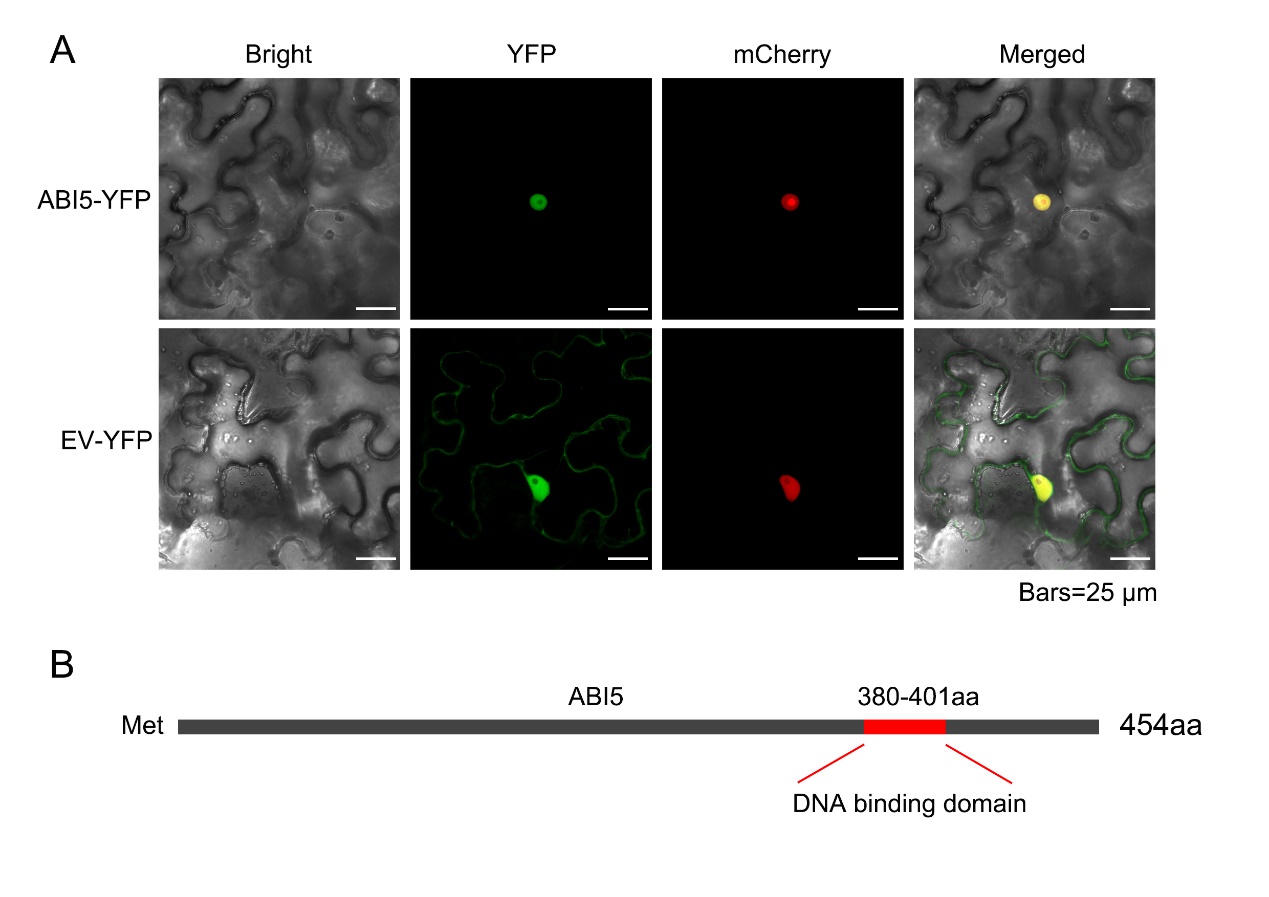


**Figure S2. The subcellular localization of CsABI5 and schematic representation of the protein domain.**

**(A)** CsABI5 is localized in the nucleus. 35S::*CsABI5*-YFP and 35S::YFP were transiently expressed in *N. benthamiana* leaves. AtH2B-mCherry served as a nucleus marker. The CsABI5-YFP signal overlapped with AtH2B-mCherry, indicating that CsABI5 is localized in the nucleus. Scale bars = 25 μm. **(B)** Schematic representation of the DNA-binding domain of CsABI5.


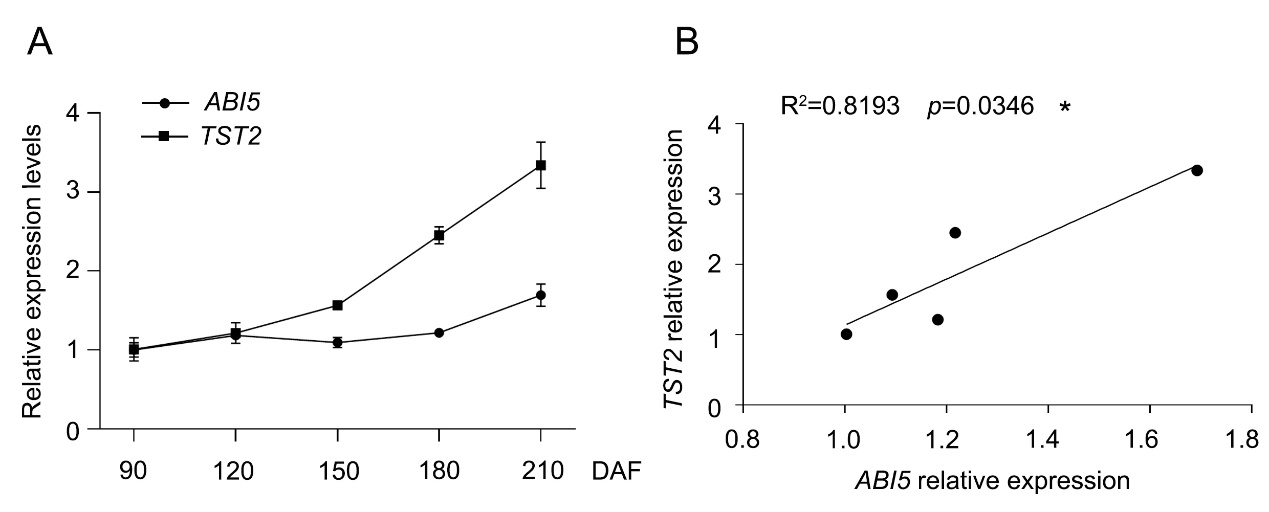


**Figure S3. Detection and correlation analysis of *CsABI5* and *CsTST2* expression during citrus fruit development stages.**

1. The transcript levels of *CsABI5* and *CsTST2* were measured during the sweet orange fruit development stages (90-210 DAF). Relative gene expression levels were quantified using *CsActin* as an internal control. The values of 90 DAF were designated as '1', and a composite sample of pulp from 9 fruits was utilized as an independent biological replicate. Bars represent the mean ± standard deviation (n = 3 independent biological replicates). **(B)** Analysis of quantified *CsABI5* and *CsTST2* expression showed that they correlate well at the transcriptional level (r² = 0.8193, * *P* < 0.05).

**
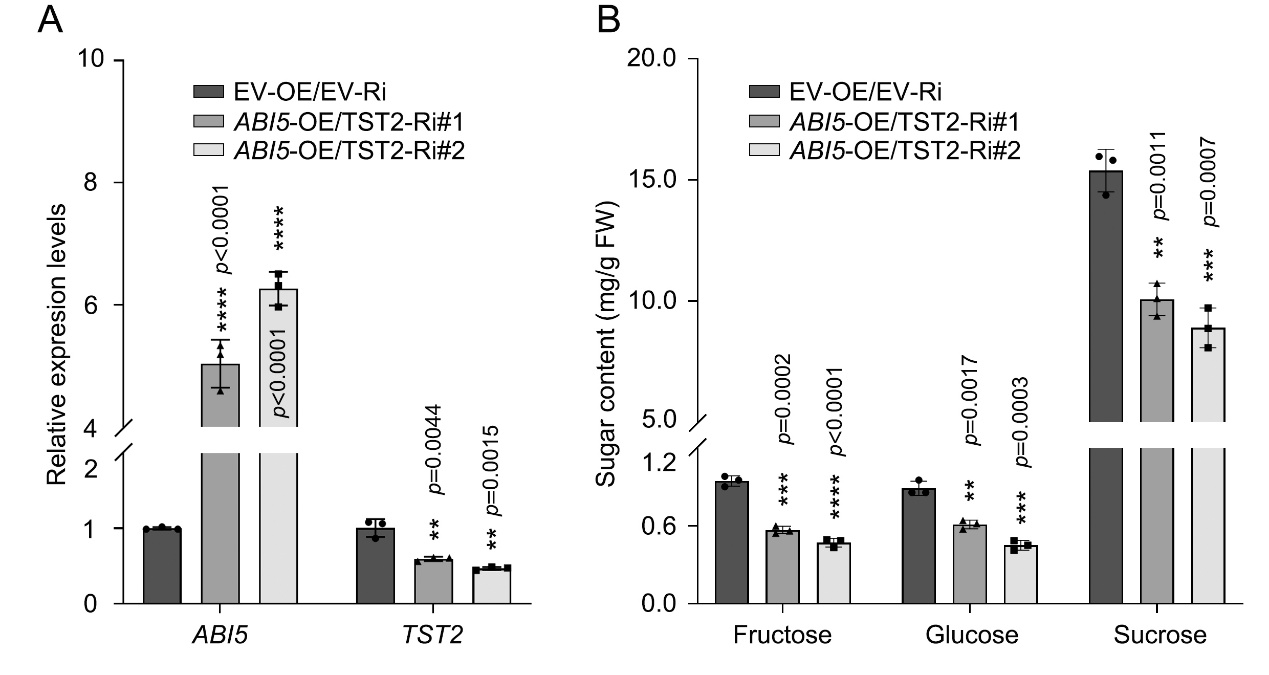
**

**Figure S4. *CsTST2* is indispensable for CsABI5-mediated the accumulation of soluble sugars.**

**(A)** Detection of *CsABI5* and *CsTST2* expression levels in *CsTST2*-silenced and *CsABI5*-overexpressed calli, with empty vector-transformed calli (EV-OE/EV-Ri) as a control, revealed that the expression levels of *CsABI5* and *CsTST2* were significantly upregulated and downregulated, respectively, with the expression of the corresponding genes in the control calli set to "1". **(B)** Compared with the control calli (EV-OE/EV-Ri), under the premise of *CsTST2* silencing, the increase in *CsABI5* expression levels failed to induce an elevation in *CsTST2* expression levels or an effective increase in soluble sugar content.


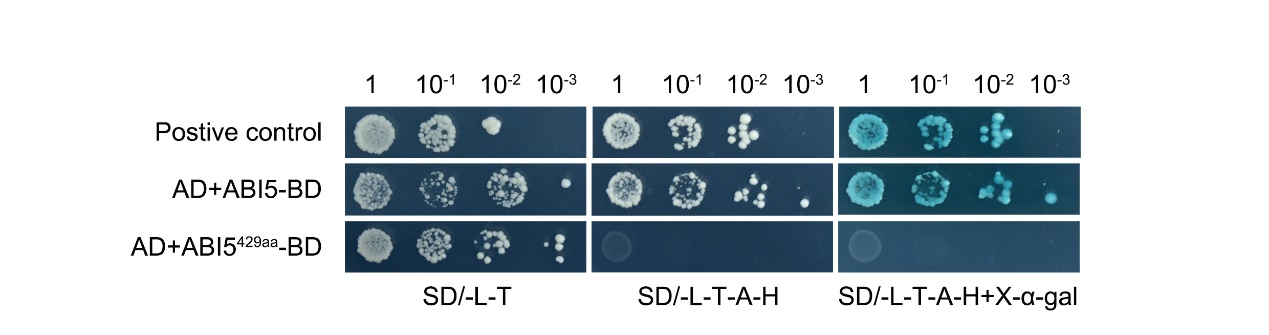


**Figure S5. Self-activation detection and elimination of the CsABI5-BD structure for yeast two-hybrid assay.**

The full length of CsABI5 presented self-activation in Y2H system. However, this self-activation was nearly entirely abolished by the removal of the C-terminal 23 amino acids of CsABI5 (CsABI5^429aa^).


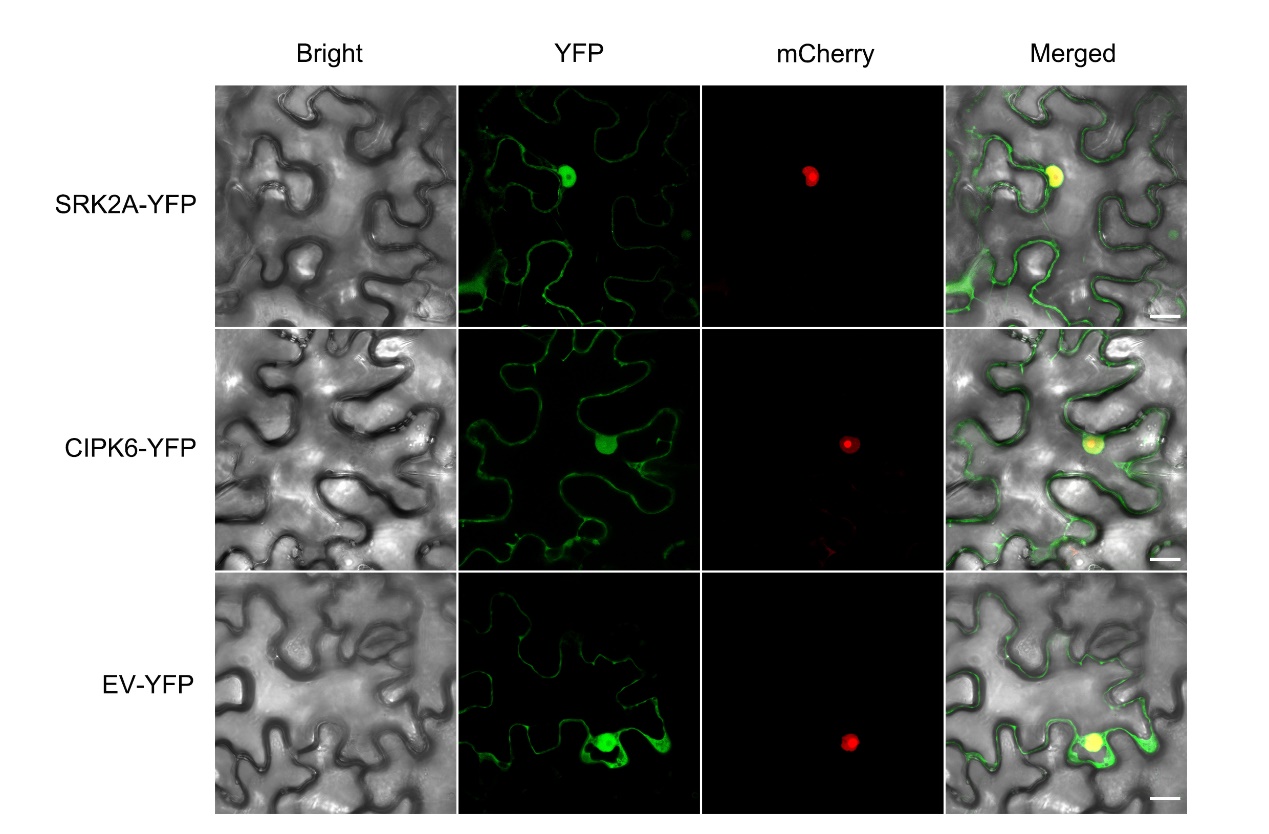


**Figure S6. The subcellular localization of CsSRK2A and CsCIPK6.**

35S::*CsSRK2A*-YFP, 35S::*CsCIPK6*-YFP and 35S::YFP empty vector were transiently expressed in *N. benthamiana* leaves. AtH2B-mCherry was utilized as a nucleus marker. Scale bars = 25 μm.


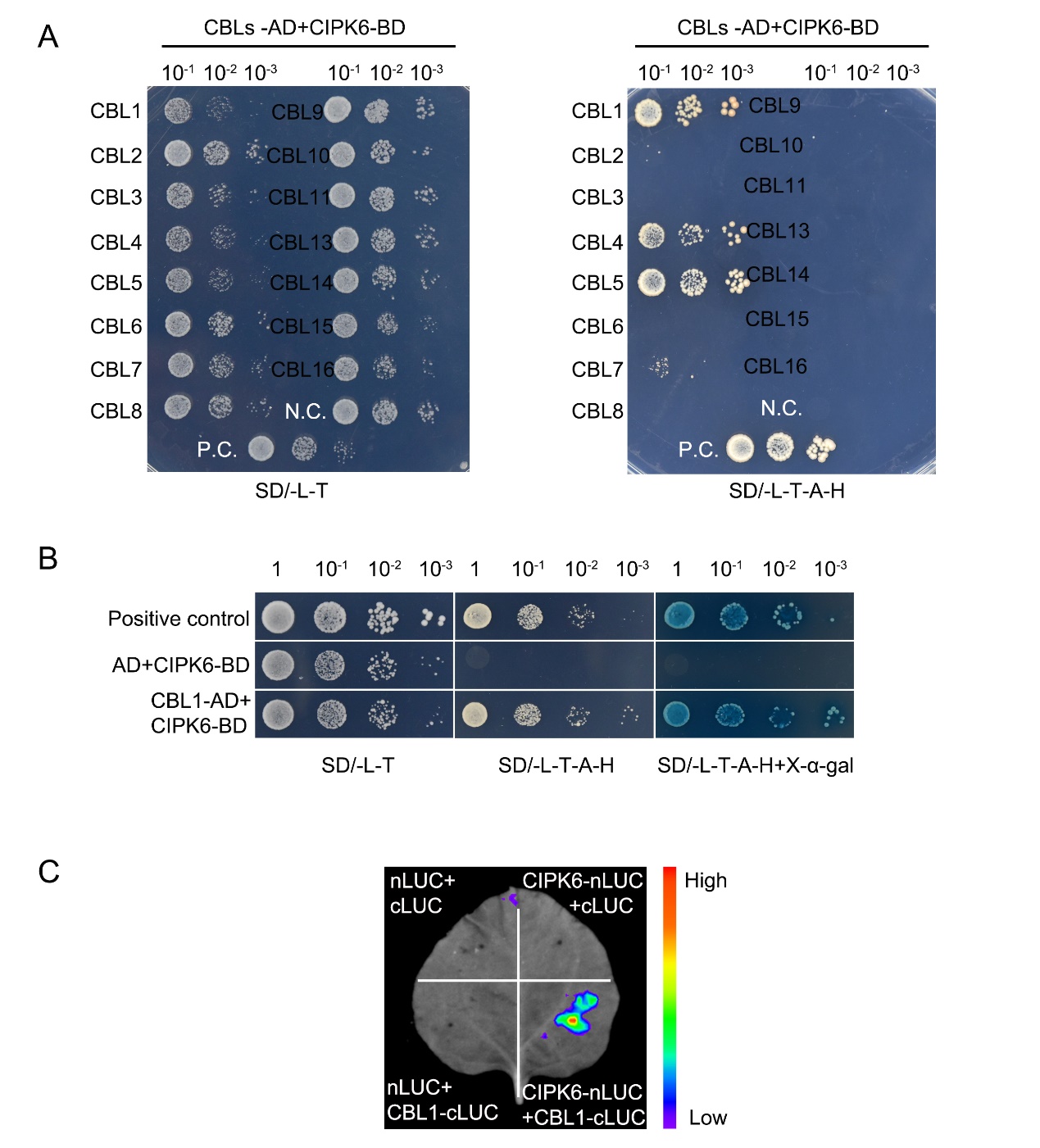


**Figure S7. The screen and further validation of CsCBL members that interact with CsCIPK6. (A)** The CDSs of fifteen *CsCBLs* were cloned into the pGADT7 vector and co-transfected into yeast Y2H-gold cells with CsCIPK6-pGBKT7, respectively. N.C.: Negative Control; P.C.: Positive Control. **(B)** Y2H point-to-point validation indicated the interaction between CsCIPK6 and CsCBL1. **(C)** The CDSs of *CsCIPK6* and *CsCBL1* were inserted into vectors containing cLUC or nLUC, respectively, and the fusion proteins were expressed in *N. benthamiana* leaves by *A. tumefaciens* GV3101-mediated transient transformation. The firefly fluorescence signal showed that CsCIPK6 interacted with CsCBL1.


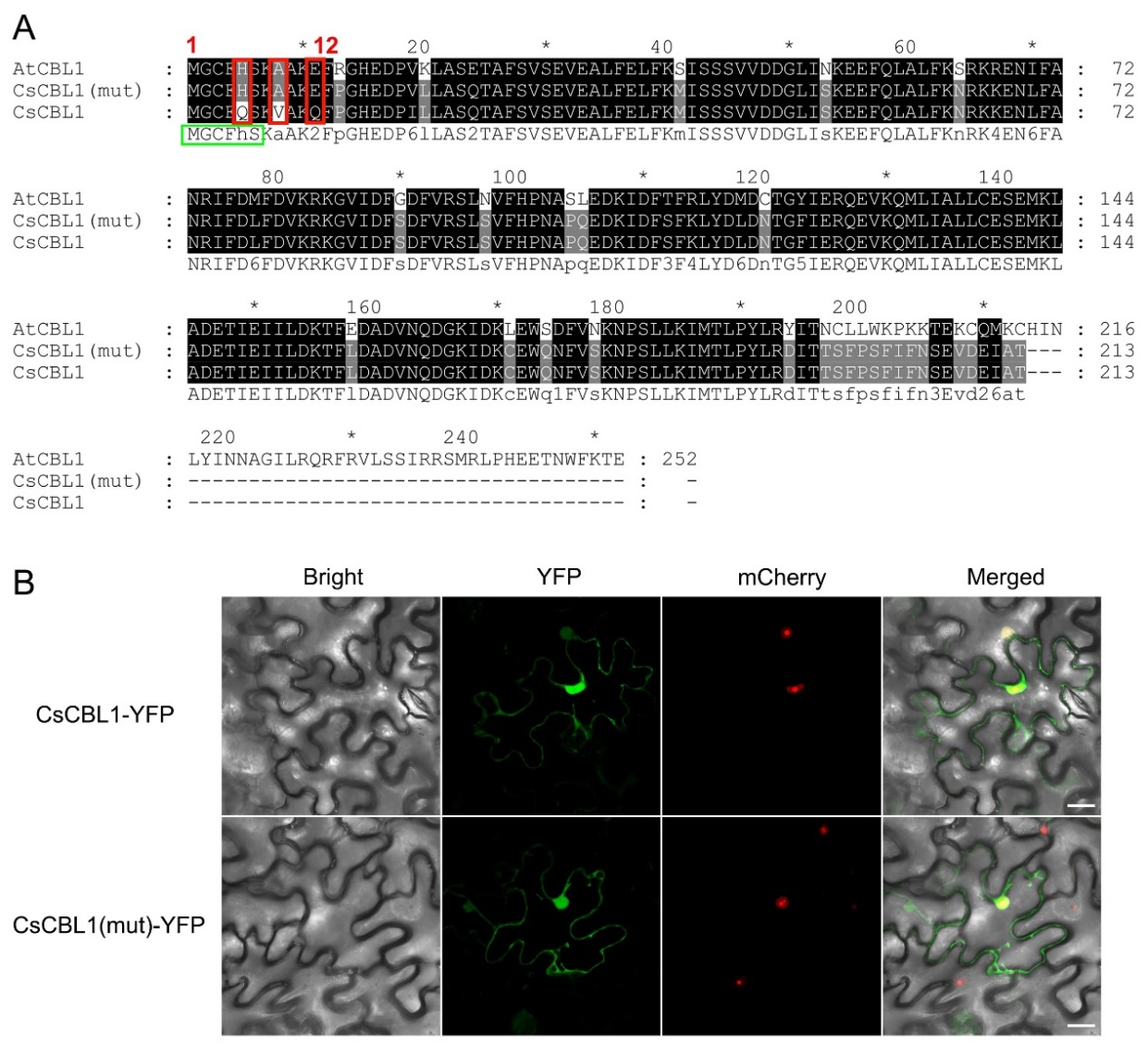


**Figure S8. The subcellular localization of CsCBL1.**

**(A)** The initial 12 amino acids of CsCBL1 were substituted with the corresponding amino acids 1-12 from Arabidopsis AtCBL1, resulting in the construct CsCBL1(mut). The red rectangles denote the positions of amino acid substitutions, while the green rectangles indicate the site of myristoylation (M-G-C-X-X-S). **(B)** The constructs 35S::*CsCBL1*-YFP and 35S::*CsCBL1*(mut)-YFP were transiently expressed in *N. benthamiana* leaves. The localization of the YFP fusion proteins in both constructs was consistent with each other. AtH2B-mCherry was utilized as a nucleus marker. Scale bars = 25 μm.


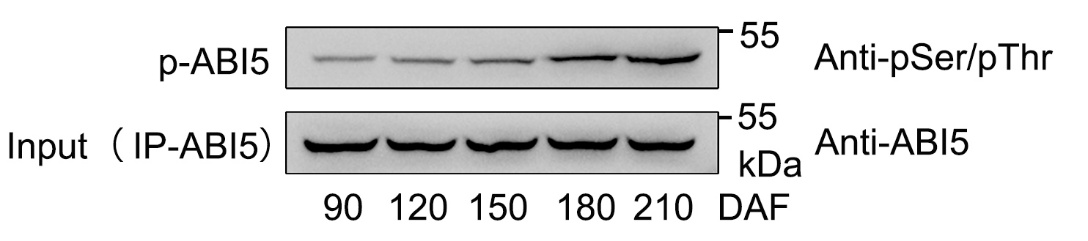


**Figure S9. Detection of the phosphorylation level of CsABI5 during fruit development.**

CsABI5 proteins in pulp tissues at different developmental stages were immunoprecipitated using specific antibodies for CsABI5. Under the premise of ensuring consistent total amounts of CsABI5, their phosphorylation level were detected using anti-pSer/pThr phosphorylation antibodies. The phosphorylation level of CsABI5 gradually increased with fruit development.


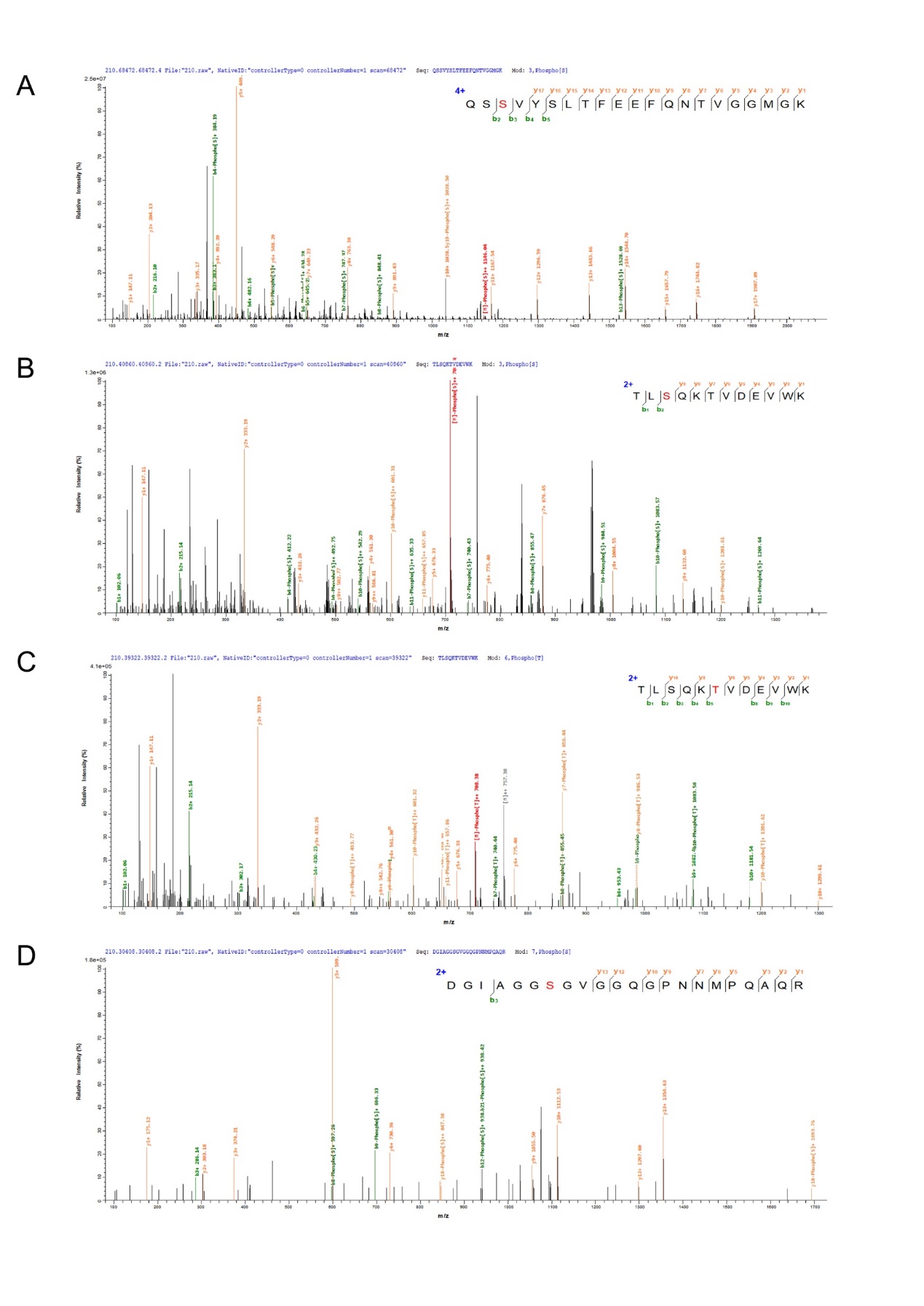


**Figure S10. Amino acid residues of CsABI5 phosphorylated by CsSRK2A were analyzed by LC-MS/MS.**

**(A-D)** Ser40 **(A)**, Ser109 **(B)**, Thr112 **(C)**, and Ser134 **(D)** residues of CsABI5 were identified as phosphorylation sites by CsSRK2A through LC-MS/MS assay. Phosphorylated Ser or Thr residues in CsABI5 are highlighted in red.


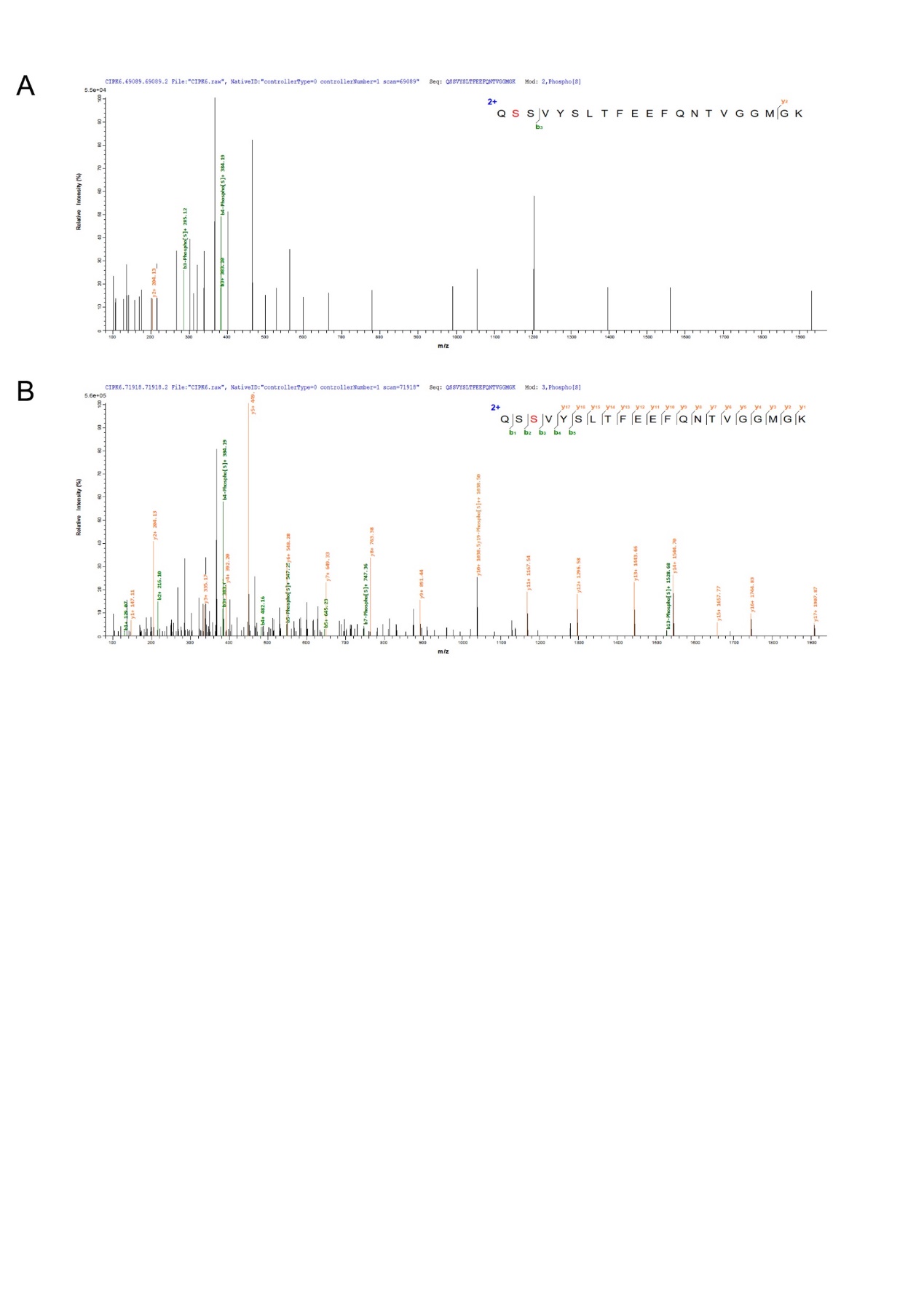


**Figure S11. Amino acid residues of CsABI5 phosphorylated by CsCIPK6 were analyzed by LC-MS/MS.**

**(A-B)** Ser39 **(A)**, Ser40 **(B)** residues of CsABI5 were identified as phosphorylation sites by CsCIPK6 through LC-MS/MS assay. Phosphorylated Ser residues in CsABI5 are highlighted in red.


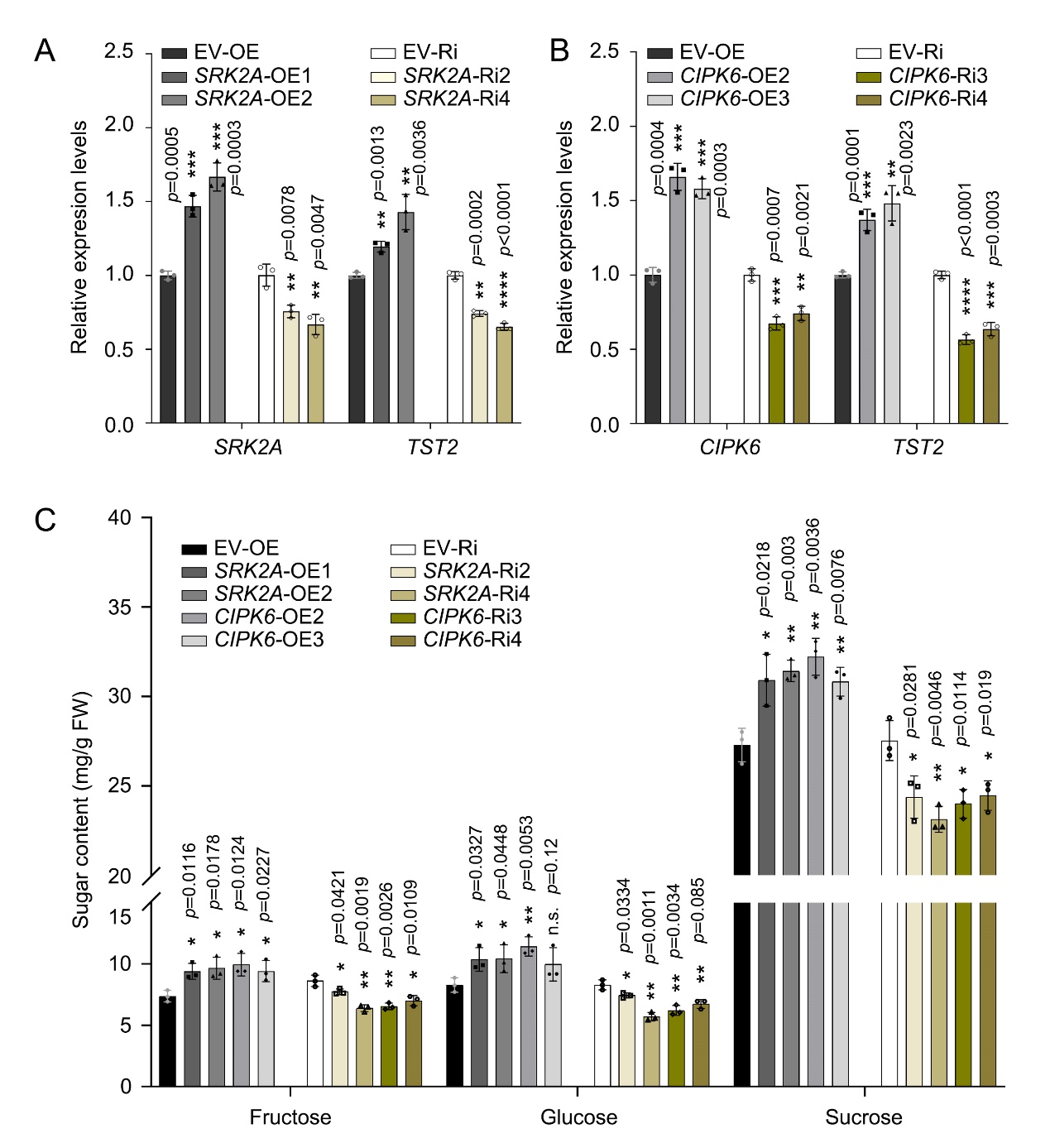


**Figure S12. Effect of transient overexpression of *CsSRK2A* or *CsCIPK6* in citrus juice sacs on *CgTST2* expression and sugar content.**

**(A-B)** Transient overexpression or silence of *CsSRK2A* (A) or *CsCIPK6* (B) in citrus juice sacs resulted in elevated or decreased *CgTST2* expression. **(C)** Transient overexpression or silence of *CsSRK2A* or *CsCIPK6* in juice sacs results in a simultaneous increase or decrease in fructose, glucose and sucrose content. The juice sacs transformed with empty vector were used as a control. Bars represent mean ± sd (n = 3 independent biological replicates). Asterisks indicate significant differences by two-tailed Student’s t-test (* *P* < 0.05, ** *P* < 0.01, *** *P* < 0.001). n.s., not significant.


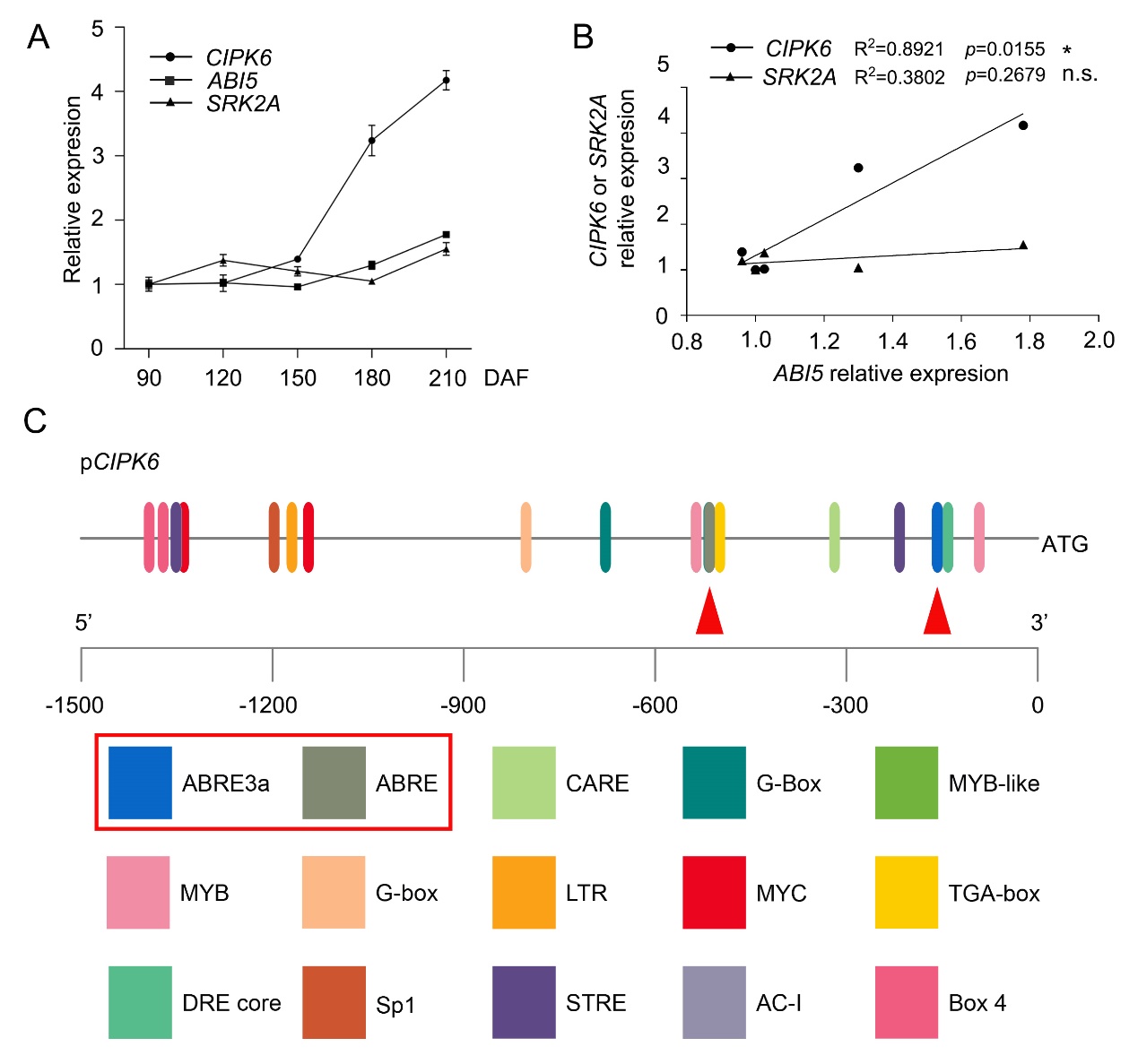


**Figure S13. Correlation analysis of *CsABI5* expression with *CsSRK2A*, *CsCIPK6* and analysis of *cis*-acting elements in the CsCIPK6 promoter.**

**(A)** The expression levels of *CsABI5*, *CsSRK2A* and *CsCIPK6* during citrus fruit development (90-210 DAF). *CsActin* works as an internal standard. The transcript levels of the three genes at 90 DAF were set to ‘1’. **(B)** Correlation analyses were performed on the expression levels of quantified *CsABI5*, *CsSRK2A* and *CsCIPK6*.The results showed that there was a good correlation between the expression of *CsABI5* and *CsCIPK6* (r² = 0.8921, * P = 0.0155), whereas no such correlation existed with the expression of CsSRK2A (r² = 0.3802, n.s., not significant, P = 0.2679). **(C)** The promoter of *CsCIPK6* was identified to contain two classical ABRE elements through Plant CARE online platform (<https://bioinformatics.psb.ugent.be/webtools/plantcare/html/>).

**Supplemental Table S1. The 113 genes co-expressed with *CsTST2* in the STEM co-expression analysis module.**

**Supplemental Table S2. Potential interaction proteins with CsABI5 through yeast two-hybrid screening.**

**Supplemental Table S3. List of primers used in this study.**
